# Effect of generalised access to early diagnosis and treatment and targeted mass drug administration on *Plasmodium falciparum* malaria in Eastern Myanmar: an observational study of a regional elimination programme

**DOI:** 10.1016/S0140-6736(18)30792-X

**Published:** 2018-05-12

**Authors:** Jordi Landier, Daniel M Parker, Aung Myint Thu, Khin Maung Lwin, Gilles Delmas, François H Nosten, Malaria Elimination Task Force Group, Chiara Andolina, Ricardo Aguas, Saw Moe Ang, Ei Phyo Aung, Naw Baw Baw, Saw Aye Be, Saw B'Let, Hay Bluh, Craig A. Bonnington, Victor Chaumeau, Miasa Chirakiratinant, Win Cho Cho, Peter Christensen, Vincent Corbel, Nicholas PJ Day, Saw Hsa Dah, Gilles Delmas, Mehul Dhorda, Arjen M Dondorp, Jean Gaudart, Gornpan Gornsawun, Warat Haohankhunnatham, Saw Kyaw Hla, Saw Nay Hsel, Gay Nay Htoo, Saw Nay Htoo, Mallika Imwong, Saw John, Ladda Kajeechiwa, Lily Kereecharoen, Praphan Kittiphanakun, Keerati Kittitawee, Kamonchanok Konghahong, Saw Diamond Khin, Saw Win Kyaw, Jordi Landier, Clare Ling, Khin Maung Lwin, Khine Shwe War Lwin, Naw K' Yin Ma, Alexandra Marie, Cynthia Maung, Ed Marta, Myo Chit Minh, Olivo Miotto, Paw Khu Moo, Ku Ler Moo, Merry Moo, Naw Na Na, Mar Nay, François H. Nosten, Suphak Nosten, Slight Naw Nyo, Eh Kalu Shwe Oh, Phu Thit Oo, Tun Pyit Oo, Daniel M. Parker, Eh Shee Paw, Choochai Phumiya, Aung Pyae Phyo, Kasiha Pilaseng, Stéphane Proux, Santisuk Rakthinthong, Wannee Ritwongsakul, Kloloi Salathibuphha, Armon Santirad, Sunisa Sawasdichai, Lorenz von Seidlein, Paw Wah Shee, Paw Bway Shee, Decha Tangseefa, Aung Myint Thu, May Myo Thwin, Saw Win Tun, Chode Wanachaloemlep, Lisa J White, Nicholas J White, Jacher Wiladphaingern, Saw Nyunt Win, Nan Lin Yee, Daraporn Yuwapan

**Affiliations:** a Shoklo Malaria Research Unit, Mahidol-Oxford Tropical Medicine Research Unit, Faculty of Tropical Medicine, Mahidol University, Mae Sot, Thailand; b Institut de Recherches pour le Développement, Aix Marseille Univ, INSERM, SESSTIM, Sciences Economiques & Sociales de la Santé & Traitement de l'Information Médicale, Marseille, France; c Department of Population Health and Disease Prevention, University of California, Irvine, CA, USA; d Centre for Tropical Medicine and Global Health, Nuffield Department of Medicine, University of Oxford, Oxford, UK

## Abstract

**Background:**

Potentially untreatable *Plasmodium falciparum* malaria threatens the Greater Mekong subregion. A previous series of pilot projects in Myanmar, Laos, Cambodia, and Vietnam suggested that mass drug administration was safe, and when added to provision of early diagnosis and treatment, could reduce the reservoir of *P falciparum* and interrupts transmission. We examined the effects of a scaled-up programme of this strategy in four townships of eastern Myanmar on the incidence of *P falciparum* malaria.

**Methods:**

The programme was implemented in the four townships of Myawaddy, Kawkareik, Hlaingbwe, and Hpapun in Kayin state, Myanmar. Increased access to early diagnosis and treatment of malaria was provided to all villages through community-based malaria posts equipped with rapid diagnostic tests, and treatment with artemether–lumefantrine plus single low-dose primaquine. Villages were identified as malarial hotspots (operationally defined as >40% malaria, of which 20% was *P falciparum*) with surveys using ultrasensitive quantitative PCR either randomly or targeted at villages where the incidence of clinical cases of *P falciparum* malaria remained high (ie, >100 cases per 1000 individuals per year) despite a functioning malaria post. During each survey, a 2 mL sample of venous blood was obtained from randomly selected adults. Hotspots received targeted mass drug administration with dihydroartemisinin–piperaquine plus single-dose primaquine once per month for 3 consecutive months in addition to the malaria posts. The main outcome was the change in village incidence of clinical *P falciparum* malaria, quantified using a multivariate, generalised, additive multilevel model. Malaria prevalence was measured in the hotspots 12 months after mass drug administration.

**Findings:**

Between May 1, 2014, and April 30, 2017, 1222 malarial posts were opened, providing early diagnosis and treatment to an estimated 365 000 individuals. Incidence of *P falciparum* malaria decreased by 60 to 98% in the four townships. 272 prevalence surveys were undertaken and 69 hotspot villages were identified. By April 2017, 50 hotspots were treated with mass drug administration. Hotspot villages had a three times higher incidence of *P falciparum* at malarial posts than neighbouring villages (adjusted incidence rate ratio [IRR] 2·7, 95% CI 1·8–4·4). Early diagnosis and treatment was associated with a significant decrease in *P falciparum* incidence in hotspots (IRR 0·82, 95% CI 0·76–0·88 per quarter) and in other villages (0·75, 0·73–0·78 per quarter). Mass drug administration was associated with a five-times decrease in *P falciparum* incidence within hotspot villages (IRR 0·19, 95% CI 0·13–0·26). By April, 2017, 965 villages (79%) of 1222 corresponding to 104 village tracts were free from *P falciparum* malaria for at least 6 months. The prevalence of wild-type genotype for *K13* molecular markers of artemisinin resistance was stable over the three years (39%; 249/631).

**Interpretation:**

Providing early diagnosis and effective treatment substantially decreased village-level incidence of artemisinin-resistant *P falciparum* malaria in hard-to-reach, politically sensitive regions of eastern Myanmar. Targeted mass drug administration significantly reduced malaria incidence in hotspots. If these activities could proceed in all contiguous endemic areas in addition to standard control programmes already implemented, there is a possibility of subnational elimination of *P falciparum*.

**Funding:**

The Bill & Melinda Gates Foundation, the Regional Artemisinin Initiative (Global Fund against AIDS, Tuberculosis and Malaria), and the Wellcome Trust.

## Introduction

The emergence and spread of artemisinin-resistant *Plasmodium falciparum* in the Greater Mekong subregion, followed by the failure of artemisinin combination therapies (ACTs) presents a serious and imminent threat to the region and beyond.^1^, ^2^, ^3^, ^4^ The countries in the Greater Mekong subregion are committed to malaria elimination in the near future,^5^ provided the available tools remain effective.

Among the many factors required for malaria elimination, effective vector control (eg, long-lasting insecticidal nets) and access to effective community-based early diagnosis and treatment stand out as essential^6^, ^7^ but these two interventions are not sufficient everywhere. In the Greater Mekong subregion, long-lasting insecticidal nets provide incomplete protection because of the biting behaviour of anopheline vectors,^8^, ^9^ and early diagnosis and treatment does not address the problem of asymptomatic parasite carriers who are an important and increasingly reported source of malaria in low transmission areas.^10^, ^11^, ^12^, ^13^ The treatment of asymptomatic carriers could therefore be important to accelerate the elimination of malaria. The poor sensitivity of point-of-care tests limits the use of active screening and treatment approaches to detect and cure asymptomatic infections. Other options to eliminate the asymptomatic, submicroscopic parasite reservoirs include risk-targeted presumptive treatment, seasonal malaria chemoprevention in high-risk groups, and mass drug administration.^14^

**Research in context**

**Evidence before this study**

In September, 2017, we searched the PubMed database for articles published since January, 2000, on *Plasmodium falciparum* or *Plasmodium vivax* malaria elimination or eradication, using the search terms ((malaria, falciparum OR malaria, vivax [MeSH Terms]) AND (“2000”[Date - Publication]: “3000”[Date - Publication]) AND (elimination, disease OR eradication, disease [MeSH Terms] OR elimination OR eradication)). Of 1249 articles, we excluded 720 articles that did not report public health or community based interventions or data (eg, on pharmacokinetics, parasite genetics or genomics, antimalarial resistance, new diagnostic tools or treatments, or protocols); 257 reviews, opinion-articles, or general policy briefs; and 70 mathematical modelling reports. Of the remaining 202 articles, 130 did not report a specific intervention; 38 of these 130 included reports of individual risk-factors or clustering of clinical or asymptomatic malaria infections. 63 articles presented specific interventions or a mixed strategy as part of a national or subnational elimination programme, including vector control (long-lasting insecticidal nets, indoor residual spraying, and larvae control); increased access to diagnosis and treatment (artemisinin combination therapy [ACT] and community-based approaches); and active case detection and mass drug administration. 40 articles reported a quantitative measurement of the effect of the intervention, such as comparisons between intervention or comparisons before and after the intervention. In terms of interventions targeting the human reservoir, reports on mass drug administration generally assessed its effect on subsequent incidence or prevalence of malaria, but active case-finding reports frequently limited themselves to the number of cases identified.

**Added value of this study**

A gap remains between empirical evidence from malaria-elimination interventions or programmes undertaken in the ACT era, and the number of available reviews, opinion pieces, and predictive models of malaria elimination. Here, we provide empirical evidence of the effect of a programme to locally eliminate malaria over a remote, difficult-to-access region in Myanmar (population >365 000) where artemisinin-resistant *P falciparum* malaria is prevalent. We quantified the overall effect of two key interventions: increased access to malaria diagnosis and treatment, and targeted mass drug administration, and showed that early diagnosis and effective treatment substantially decreased village-level incidence of *P falciparum* malaria.

**Implications of all the available evidence**

This study adds to other evidence confirming that at the subnational level, an elimination strategy based on the provision of early diagnosis and treatment can substantially decrease *P falciparum* incidence, and suggests that a few targeted mass drug administration interventions had a strong effect on high prevalence reservoirs of *P falciparum*.

A series of pilot projects assessing mass drug administration with dihydroartemisinin and piperaquine plus single-dose primaquine were done in Myanmar, Laos, Cambodia, and Vietnam between 2013 and 2016.^15^, ^16^, ^17^ These studies suggested that targeted mass drug administration was safe and feasible, and that when added to the provision of early diagnosis and treatment, mass drug administration could reduce the reservoir of *P falciparum* by 90% in three months and thereby interrupt malaria transmission.^15^, ^17^ Questions remained, however, about the scalability of this approach.^18^ The objectives of the programme described here were to scale-up this novel strategy regionally and measure its effect on the incidence of clinical malaria in eastern Karen/Kayin state, Myanmar, a difficult-to-access hilly and forested area with complex political and geographical landscapes,^8^ where malaria transmission is seasonal and where artemisinin resistant *P falciparum* is prevalent.

## Methods

### Study design and participants

This programme was implemented in four administrative subdivisions (townships appendix) of Kayin State, Myanmar (Myawaddy, Kawkareik, Hlaingbwe, and Hpapun) in partnership with eight community-based health organisations responsible for the malaria-post network in the catchment areas of their health facilities (community-based malaria clinics were termed malaria posts). Coordination and management were centralised and an executive committee including representatives of each organisation made operational decisions.

Two main interventions were deployed: access to early diagnosis and treatment through malaria posts in all villages and targeted mass drug administration in so-called hotspot villages. A village was classified as a malaria hotspot when the 90% CI upper limit of the sum of *P falciparum* and *Plasmodium vivax* prevalence estimate was at least 40% and the corresponding value of the proportion of *P falciparum* in the positive samples was at least 20%. Villages with prevalence below this threshold, or those not surveyed, were termed non-hotspot villages. The main outcome of the study was the incidence of clinical *P falciparum* malaria. A summary of the protocol is provided here, the detailed protocol has been published elsewhere.^19^

All individuals participating in blood surveys and mass drug administration provided written informed consent. This project was approved through the ethics review committee on medical research involving human beings from Myanmar, Ministry of Health and Sports, Department of Medical Research (lower Myanmar): 73/Ethics 2014.

### Geographic reconnaissance, community engagement, and vector control

The project began by establishing a geographic information system. The area was mapped and surveyed including village name(s), estimates of house counts, and whether current malaria services were available in the community.^19^ Community engagement activities were used to introduce and accompany all programme components. These activities were done by a team already experienced in malaria work.^20^ The main aim of the activities was to ensure community understanding, support, and ultimately ownership of the facilities and interventions provided by the programme. Over 3 years, 60 000 additional long-lasting insecticidal nets were distributed to achieve and maintain universal coverage.

### Malaria posts and malaria incidence through passive case detection

The malaria posts provided access to free early diagnosis and treatment. Trained workers diagnosed malaria infections using SD Bioline Malaria Ag *P falciparum* or *P vivax* rapid diagnostic tests (RDT (Alere/SD, South Korea) and treated uncomplicated *P falciparum* infections with an artemether–lumefantrine combined treatment (artemether 5–24 mg/kg and lumefantrine 29–144 mg/kg, orally, twice per day for 3 days), except for pregnant women in their first trimester who received quinine and clindamycin (quinine 10 mg/kg and clindamycin 5 mg/kg, orally three times per day for 7 days). All patients (excluding young infants, pregnant women, and breastfeeding mothers) received a single low dose of primaquine (0·25 mg/kg once on the first day of treatment). Pregnancy tests were offered to women unsure of their pregnancy status. *P vivax* infections were treated with chloroquine (over 3 days, 10 mg/kg on days 1 and 2, and 5 mg/kg on day 3). All severe cases were referred to the nearest health-care facility, such as a village tract health centre or clinic (primary level) or township hospital (secondary level). Medicines and RDT were supplied by the National Malaria Control Programme supported by The Global Fund. Malaria-post workers reported weekly the numbers of fever cases, RDT-confirmed infections, and treated *P falciparum* and *P vivax* malaria cases. Data were transmitted using either a smartphone application or on paper carried by messengers. Activity monitoring and malaria surveillance were done weekly using the reported data. Confirmed malaria cases, diagnosed by RDT in malaria posts, hereafter referred to as clinical cases of either *P falciparum* or *P vivax*, were the basis of incidence estimates.

### Definition and detection of hotspots to identify MDA sites

The operational definition of a malaria hotspot was based on previous studies in the region using ultrasensitive PCR.^15^, ^16^ Infections confirmed by ultrasensitive PCR, hereafter referred to as *P vivax* or *P falciparum* infections rather than clinical cases, were the basis of prevalence estimates in this work as well. To identify hotspots and investigate spatial patterns in prevalence, surveys originally targeted randomly selected villages.^19^ The sample size was calculated to detect a 40% malaria prevalence with plus or minus 10% precision of a 90% CI with 80% power. After 1 year (approximately 100 surveys) it became apparent that hotspot villages clustered spatially; subsequent surveys systematically included neighbouring villages, within 10 km of an identified hotspot. Villages where the incidence of clinical cases of *P falciparum* malaria remained high (>100 cases per 1000 individuals per year) despite a functioning malaria post were also targeted by a prevalence survey. During each survey, a 2 mL sample of venous blood was obtained from randomly selected adults^20^ after individual informed consent. Blood samples were transported to the laboratory within 24h to 48h of collection and analysed using ultrasensitive PCR, with a lower limit of detection of 22 parasites per mL.^21^ Individuals with *Plasmodium* infections confirmed with ultrasensitive PCR were not treated unless they had fever (axillary temperature >37·5°C) and were RDT-positive during the survey.

### Mass drug administration in hotspots and follow-up surveys

Mass drug administration consisted of three consecutive rounds administered 1 month apart.^17^ At each round a weight-based course of dihydroartemisinin–piperaquine (dihydroartemisinin 7 mg/kg and piperaquine 55 mg/kg orally, once per day for 3 days). Children younger than 6 months, individuals with a known allergy to the antimalarial drugs, and women in the first trimester of pregnancy were excluded from receiving the MDA regimen. Pregnant women in their second or third trimester and breastfeeding mothers were eligible for dihydroartemisinin–piperaquine but not primaquine. During the mass drug administration, a mobile clinic operated by the team offered medical care to all villagers and ensured passive recording of adverse events. If not already present, a malaria post was established in the village before the start of the mass drug administration intervention and continued functioning after its end. Follow-up surveys were done in mass drug administration villages at 12 months and later after mass drug administration following the same procedures. The sample size of the malarial surveys at 12 months was calculated to detect with 80% power a 90% decrease from baseline,^17^ and to achieve a precision of the 95% CI lower than the expected prevalence value (ie, for a 15% baseline falciparum prevalence, the expected prevalence of malaria at 12 months was 1·5% and the sample size aimed at 95% CI 0–3%).^19^

### Surveillance of genetic markers of drug resistance

The surveillance of drug resistance markers was done on positive RDTs or on dried blood spots from *P falciparum* clinical cases collected by malaria posts, and on dried blood spots from *P falciparum* RDT-positive individuals collected during surveys. Parasite DNA was extracted from *P falciparum*-positive RDTs and dried blood spots to detect the presence of genetic markers of *P falciparum* antimalarial resistance. Markers included *PfKelch13* mutations for artemisinin resistance, *Pfmdr1* amplification for mefloquine, and plasmepsin 2 or 3 amplification for piperaquine resistance.^19^

### Statistical analysis

Statistical analysis was done using Stata v14.1, R v3.4.0, R-package mgcv, and the Spatial Analysis in Macroecology software package (v 4.0).

Incidence rates (cases per 1000 population per unit of time) and Poisson 95% CIs were calculated from weekly malaria post reports. Cumulative incidence rates were calculated for different aggregate units of space and time: by week, month, or year; and by village, village tract (the smallest administrative unit in Myanmar [appendix]), or township. The denominator (person-time exposed) was calculated at village level as the village population multiplied by the number of weekly reports over the period of interest, then summed over the spatial unit of interest. Yearly incidence of clinical *P falciparum* and *P vivax* was aggregated by village tract for visual analysis. Missing reports were rare (appendix) and omitted.

A generalised, additive, multilevel, mixed negative binomial model was used to measure the effect on village-level incidence of *P falciparum* of early diagnosis and treatment, mass drug administration, and the coverage of early diagnosis and treatment within the village tract. Adjustments were included for seasonality and location (latitude, longitude, and altitude). Village population size (natural log-transformed) was included as an offset and random effects terms (for intercept and slope) were used at the village level to account for unexplained local heterogeneity and repeated measures by village. Different functional forms were used to model the effect of early diagnosis and treatment over time (appendix). Its effect on clinical *P vivax* incidence was also assessed using the same model and set of variables. The size of the malaria reservoirs was quantified by the prevalence of *P falciparum* or *P vivax* (ie, the proportion of positive results) with a Wilson binomial 95% CI (corrected for finite population size), calculated using ultrasensitive PCR survey results.

Spatial patterns in village-level prevalence, reservoir hotspots, and yearly incidence were assessed using exploratory spatial analysis including spatial correlograms. Progress towards achieving zero-incidence locally was measured using the time interval between the opening of a malaria post in a village or a village tract and the last case of *P falciparum* malaria recorded in the village or village tract. To account for stochastic and seasonal fluctuations in malaria incidence in small population sizes, malaria posts, or village tracts with at least 6 months follow-up since the last reported clinical case of *P falciparum* were considered to have reached their last case before the censoring date on April 30, 2017. Villages with fewer than 6 months follow-up since the last reported case of *P falciparum* were considered still endemic.

### Role of the funding source

The funders of the study had no role in study design, data collection, data analysis, data interpretation, or writing of the report. JL, DMP, AMT, KML, GD, and FHN had access to all the data in the study and had final responsibility for the decision to submit for publication.

## Results

Between May 1, 2014, and April 30, 2017, 1222 malaria posts were opened (82% of the total 1490 villages mapped), providing early diagnosis and treatment to an estimated 365 000 individuals. The deployment of the malaria post network was stepwise, moving from east (close to the border with Thailand) to west and eventually including villages in 157 rural village tracts, covering an estimated 18 002 km^2^ (excluding urban areas; appendix). The median number of villages per village tract was six (IQR 3–10, n=157) and the median population was 260 inhabitants per village (140–480, n=1222; (table 1). From May 1, 2014, to April 30, 2017, 238 682 individuals with fever were tested, resulting in the diagnosis and treatment of 10 791 cases of *P falciparum* and 17 490 cases of *P vivax* (table 2). Confirmed malaria cases represented 12% of all febrile illnesses. Patients with malaria presented at the malaria post after a median self-reported delay of 1 day since fever onset (IQR 1–2).

**Table 1:** Summary of the deployment by main administrative divisions (townships) from May, 2014, to April, 2017

	**May, 2014–April, 2015**	**May, 2015–April, 2016**	**May, 2016–April, 2017**	**Total**
Cumulative number of villages equipped with malaria posts at the end of the period (total corresponding population)	487 (144 100)	879 (240 620)	1222 (365 000)	1222 (365 000)
Total area covered (km^2^; cumulative)	15 715	16 826	18 002	18 002
Number of surveys done (targeted surveys)	140 (21)	87 (8)	45 (36)	272 (65)
Number of hotspots identified (by targeted surveys)	29 (7)	18 (6)	22 (21)	69 (34)
Number of MDAs done	11	32	7	50*
Number of M12 surveys	0	11	29	40

MDA=mass drug administration. M12=malaria prevalence 12 months after MDA.

* 19 remaining hotspots unaddressed by April 30, 2017.

**Table 2:** Changes in malaria yearly cumulative incidence by main administrative divisions (townships) from May, 2014, to April, 2017

	**May, 2014, to April, 2015**		**May, 2015, to April, 2016**		**May, 2016, to April, 2017**		**May, 2014, to April, 2017 crude decrease (%)**
	Number of cases/person–years	IR (95% CI) cases per 1000/year	Number of cases/person–years	IR (95% CI) cases per 1000/year	Number of cases/person–years	IR (95% CI) cases per 1000/year	
***Plasmodium falciparum* cumulative incidence**							
Hpapun	1698/17 783	95·5 (90·8–100·0)	3606/61 308	58·8 (56·9–60·8)	3186/84 201	37·8 (36·5–39·2)	60%
Hlaingbwe	37/11 305	3·3 (2·3–4·5)	75/46 283	1·6 (1·3–2·0)	86/143 610	0·6 (0·5–0·7)	82%
Kawkareik	22/4360	5·0 (3·2–7·6)	46/25 852	1·8 (1·3–2·4)	31/96 510	0·3 (0·2–0·5)	94%
Myawaddy	520/35 163	14·8 (13·5–16·1)	117/50 717	2·3 (1·9–2·8)	18/56 679	0·3 (1·2–0·5)	98%
***Plasmodium vivax* cumulative incidence**							
Hpapun	1695/17 783	95·3 (90·8–100·0)	3093/61 308	50·4 (48·7–52·3)	3595/84 201	42·7 (41·3–44·1)	55%
Hlaingbwe	300/11 305	26·5 (23·6–29·7)	389/46 283	8·4 (7·6–9·3)	418/143 610	2·9 (2·6–3·2)	89%
Kawkareik	90/4360	20·6 (16·6–25·4)	181/25 852	7·0 (6·0–8·1)	148/96 510	1·5 (1·3–1·8)	93%
Myawaddy	2184/35 163	62·1 (59·5–64·5)	2444/50 717	48·2 (46·3–50·1)	1670/56 679	29·5 (28·1–30·9)	52%

Coverage area was calculated using 15 km radius buffers around geographical coordinates of the community-based malaria posts, clipped using administrative boundaries around the Malaria Elimination Task Force area so that the buffer was not allowed to extend into areas not within the target area (ie, Thailand or the Thandaung township). IR=incidence rate.

50 hotspots received mass drug administration in five campaigns (from January, 2015, to December, 2016; appendix). Mass drug administration targeted a 12 465 individuals (approximately 3% of the target area population). The median proportion of village populations taking at least one round of mass drug administration was 91% (IQR 86–95, n=50 villages), and taking three rounds was 64% (50–78). No serious adverse reactions were observed through passive reporting. Details of the programme roll-out are in the appendix.

**Figure 1:**
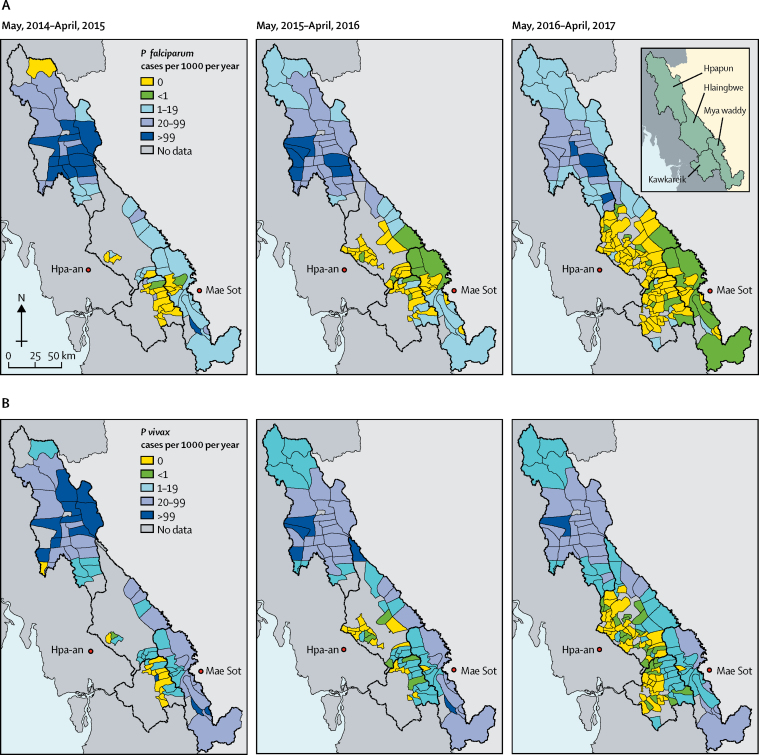
Incidence of (A) *Plasmodium falciparum* and (B) *Plasmodium vivax* over 3 years of the programme Data are the number of cases per 1000 individuals per year by village tract (lowest administrative division).

Baseline malaria incidence was markedly heterogeneous at both township and village tract levels. Seasonality patterns differed between the northern township of Hpapun (incidence peaks in June [start of rainy season] and December [cold season]) and the other townships (incidence peak in June only; appendix). The decrease in malaria incidence was also heterogeneous (figure 1). Between year 1 (May, 2014–April, 2015) and year 3 (May, 2016–April, 2017), the crude cumulative incidence of *P falciparum* malaria decreased in the four townships (figure 1, table 1, appendix). The incidence of *P vivax* decreased as well (figure 1, table 1), but seasonal fluctuation in *P vivax* incidence persisted in Hpapun and Myawaddy (appendix).

Most *Plasmodium* infections detected by ultrasensitive PCR in surveys were undetectable by microscopy or RDT. Of 14 891 samples with results for all three tests, 603 were positive for *P falciparum*, 2003 for *P vivax*, and 206 for mixed *P falciparum* and *P vivax* by PCR. Microscopy detected 28% (226/809) of all *P falciparum* or mixed qualitative PCR-positive infections, and RDT detected 34% (273/809). Microscopy detected 12% (260/2209) of *P vivax* and mixed PCR-positive infection and RDT detected 4% (79/2209; appendix).

Baseline prevalence (proportions) of *Plasmodium* infection measured by PCR at village level was heterogeneous, with a median 21% (IQR 8–35, n=272 villages). The median *P falciparum* infection prevalence was 3% (0–11), while *P vivax* was 15% (5–25). Significant clustering of high prevalence of *P falciparum* infection occurred in villages surveyed randomly (n=207), with 60% of hotspots (21/35) located within 5 km, and 94% (33/35) within 10 km of one another (appendix).

**Figure 2:**
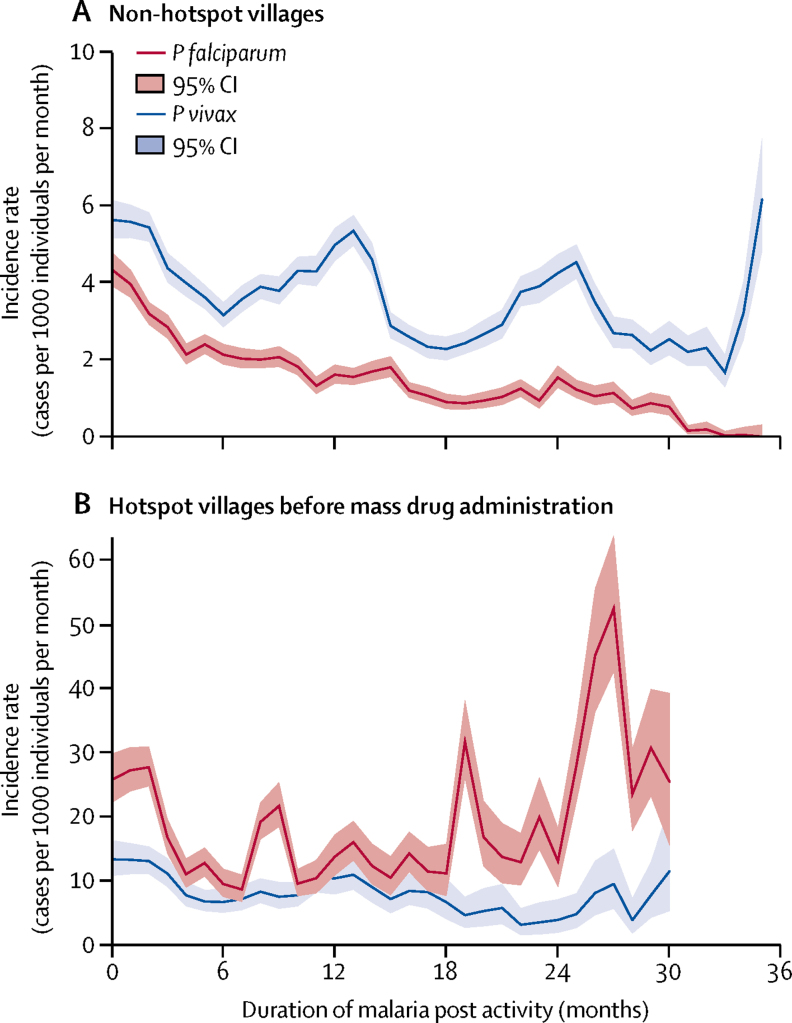
Malaria incidence since malaria post opening in the townships of Hpapun (468 malaria posts) and Myawaddy (100 malaria posts) where hotspots were identified (52 of 69 located in Hpapun Township. 7 of 69 in Myawaddy). Different y-scales are used in each graph. Falciparum in red and vivax in blue. Graphs show (A) non-hotspot villages. The widening CI after 24 months indicated that fewer malaria posts had been active for 2 years or more. The oscillations in *Plasmodium vivax* incidence are related to seasonal peaks occurring in the same locations in Myawaddy township (appendix). (B) hotspot villages before mass drug administration. Only 16 hotspots contributed to follow-up for durations of malaria post activity above 18 months. These high-incidence locations were only identified during the final campaign of baseline surveys (November, 2016, to January, 2017) and had not been addressed by April, 2017. The median follow-up before mass drug administration was 15 months (IQR 8–22), including addressed hotspots (median 12 months, IQR 5–16, n=50) and hotspots remaining unaddressed (median 32 months, 25–33, n=18). Data missing for one hotspot, which could not be equipped with a malaria post (appendix).

Setting up a malaria post in a community resulted in a decrease in the incidence of *P falciparum* clinical malaria in villages categorised as non-hotspots (figure 2). In hotspots without mass drug administration, crude *P falciparum* incidence was higher and exhibited little or no decrease even after 24 months of malaria post activity (figure 2). After adjustment for season, location, and other interventions in a multivariable analysis, the duration of malaria post activity was associated with a decrease in *P falciparum* incidence in a non-hotspot village and a slower decrease in a hotspot village before mass drug administration. Malaria post activity was associated with a slower decrease in adjusted *P vivax* incidence compared with *P falciparum*; similar in both non-hotspot and hotspot villages before mass drug administration (table 3).

**Table 3:** Effect of the interventions on incidence of clinical episodes of falciparum and vivax malaria

		**Plasmodium falciparum**			**Plasmodium vivax**		
		IRR	95%CI	p value	IRR	95%CI	p value
Type of village							
	No MDA (non-hotspot or unknown)	1	Ref		1	Ref	
	Hotspot before MDA*	2·69	1·80–4·04	<0·0001	1·87	1·32–2·67	<0·0001
	Hotspot after MDA*	0·50	0·31–0·80	<0·0001	0·48	0·32–0·73	<0·0001
Duration of malaria post activity in the village, by type of village (linear approximation, per quarter increment)							
	No MDA (non-hotspot or unknown)	0·75	0·73–0·78	<0·0001	0·93	0·91–0·94	<0·0001
	Hotspot before MDA	0·82	0·76–0·88	<0·0001	0·93	0·87–0·98	<0·0001
	Hotspot after MDA	0·99	0·92–1·06	<0·0001	1·05	1·00–1·11	<0·0001
Number of MDA done within the village tract excluding the village (per village increment)		0·96	0·92–1·00	0·0471	0·98	0·95–1·01	0·2538
Coverage of villages within the village tract (+10%)		0·90	0·87–0·93	<0·0001	0·92	0·90–0·95	<0·0001
Malaria post embedded in a community clinic		1·75	1·15–2·66	0·0087	1·44	1·04–2·00	0·0293

Data presented by the regional elimination programme. Incidence rate ratios (IRR) show the relative contribution of mass drug administration (MDA), community-based malaria posts, and coverage to the decrease in village-level and incidence after adjustment for seasonality, geographical location, and other interventions in a multivariable analysis. p values correspond to the inclusion of the three-category variable.

* Using the category hotspot before MDA as the reference, this translates to IRR 0·19 (0·13–0·26) for the hotspot after MDA.

**Figure 3:**
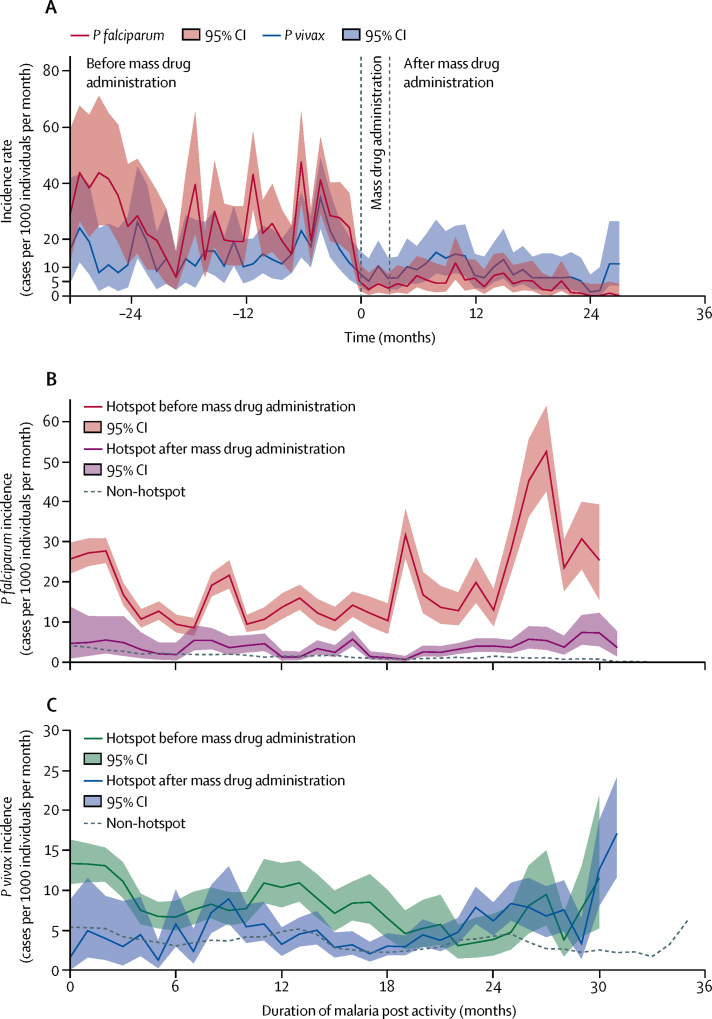
Mean *Plasmodium falciparum* and *Plasmodium vivax* incidence in hotspots before and after mass drug administration Data are (A) centred on the date of mass drug administration. Each hotspot contributes different lengths of follow-up before and after mass drug administration. The median follow-up before mass drug administration was 15 months (IQR 8–22), including addressed hotspots (median 12 months, 5–16, n=50) and hotspots remaining unaddressed (median 32 months, 25–33, n=18). The median follow-up after mass drug administration was 20 months (IQR 14–24, n=50). A marked decrease in *P falciparum* incidence after mass drug administration is observable, despite an increase in incidence around 15 months after mass drug administration. This increase is related to five hotspots (of 52 followed up to month 18) showing an incidence above 50 cases per 1000 individuals for 1 month during the second year after mass drug administration. This increase did not persist. Other graphs show incidence by duration since malaria post opening, according to before, or after, mass drug administration status for (B) *P falciparum* and (C) *P vivax*. Non-hotspot incidence trend (dashed line) is presented for reference. Different numbers of villages contributed to each estimate according to the timing of their mass drug administration.

The crude incidence of clinical *P falciparum* malaria decreased after mass drug administration compared to before, from approximately 25 cases per 1000 individuals per month to five cases per 1000 individuals per month, irrespectively of duration since the opening of the malaria post (figure 3). For *P vivax*, there was little change in incidence which remained stable around ten cases per 1000 per month (figure 3). Village-level comparisons of incidence before and after mass drug administration are in the appendix.

Adjusted *P falciparum* incidence was significantly higher in hotspots before mass drug administration than in neighbouring non-hotspot villages, and significantly lower after mass drug administration (table 3). This was equivalent to a five times decrease after mass drug administration compared with before. In hotspot villages before mass drug administration, baseline *P vivax* incidence was higher than in neighbouring non-hotspot villages. *P vivax* incidence decreased compared with neighbouring non-hotspot villages immediately after mass drug administration, but increased again afterwards.

The decrease in *P falciparum* clinical incidence after mass drug administration corresponded to a species-specific reduction of the reservoir of *Plasmodium* infections: between malaria surveys at baseline and 12 months after mass drug administration, *P falciparum* infection prevalence decreased by a median 92% (IQR 81–100, n=40) while *P vivax* prevalence decreased by a median 19% (8–47; n=40).

Geographic coverage of malaria posts within a village tract also had an effect at the village level. For each 10% increase in the proportion of villages equipped with a malaria post within a given village tract, village level *P falciparum* and *P vivax* incidence decreased by 10% (table 3). The number of additional hotspots already addressed with mass drug administration in the same village tract was associated with a small decrease in risk (table 3).

Over 3 years, a *PfKelch13* genotype result was obtained for 631 samples and the prevalence of wild-type alleles remained at 39% with a corresponding prevalence of *PfKelch13* mutants of 61% (table 4). Individual genotype trends are difficult to interpret due to the wider geographical region sampled over time. Among the mutations associated with artemisinin resistance, *C580Y* remained around 5%. An increase was observed in the proportions of other markers, also associated with artemisinin-resistance: *P441L* prevalence increased from 2% in 2014 to 13% in 2016 (table 4). Nine samples of 437 tested had multiple *Pfmdr1* copies indicative of mefloquine resistance. None of the 547 samples tested had multiple *plasmepsin2* copies; ie, there was no evidence of piperaquine resistance (table 4). The prevalence of *Pfmdr1* multiple copy numbers was 2% in 2016, compared with 60% in this region in 2013.^22^

**Table 4:** Detection of molecular markers of antimalarial resistance in rapid diagnostic tests or dried-blood spots

	**2014**		**2015**		**2016**		**Total**	
	n/N	Prevalence (%; 95% CI)	n/N	Prevalence (%; 95% CI)	n/N	Prevalence (%; 95% CI)	n/N	Prevalence (%; 95% CI)
*K13* wild type	101/258	39% (33–45)	29/81	36% (25–47)	119/292	41% (35–47)	249/631	39% (36–43)
*K13* propeller mutants	157/258	61% (55–67)	52/81	64% (53–75)	173/292	59% (53–65)	382/631	61% (57–64)
C580Y*	14/258	5% (3–9)	9/81	11% (5–20)	10/292	3% (2–6)	33/631	5% (4–7)
R561H*	24/258	9% (6–14)	2/81	2% (0–9)	14/292	5% (3–8)	40/631	6% (5–9)
R539T*	1/258	0·3% (0–2)	0/81	0% (0–4)	0/292	0% (0–1)	1/631	0·2% (0–1)
*P574L*†	4/258	2% (0–4)	6/81	7% (3–15)	0/292	0% (0–1)	10/631	2% (1–3)
*F446I*†	27/258	10% (7–15)	0/81	0% (0–4)	41/292	14% (10–19)	68/631	11% (8–13)
*P441L*†	6/258	2% (1–5)	8/81	10% (4–19)	39/292	13% (10–18)	53/631	8% (6–11)
*G449A*†	9/258	3% (2–7)	0/81	0% (0–4)	15/292	5% (3–8)	24/631	4% (2–6)
*M476I*‡	16/258	6% (4–10)	1/81	1% (0–7)	12/292	4% (2–7)	29/631	5% (3–7)
*G533S*‡	22/258	9% (5–13)	8/81	10% (4–19)	2/292	1% (0–2)	32/631	5% (3–7)
Piperaquine resistance	..	..	0/51	0% (0–7)	0/496	0% (0–0·7)	0/547	0% (0–0·7)
Mefloquine resistance	..	..	..	..	9/437	2% (1–4)	9/437	2% (1–4)

Samples were collected from individuals positive for *Plasmodium falciparum* in a rapid diagnostic test (RDT). Individuals were diagnosed at a community-based malarial post or detected during a prevalence survey. Total number of samples collected were 1241 RDT in 2014; 125 dried-blood spots (DBS) in 2015; and 949 RDT and 446 DBS in 2016. Total number of samples with at least one result were 258 (21%) in 2014; 89 (71%) in 201; and 175 RDT (18%) and 333 DBS (75%) of 508 in 2016. ..=data unavailable. Selected *K13* propeller mutants:

* validated for artemisinin-resistance;

† candidate for artemisinin-resistance;

‡ other *K13* polymorphisms. The variations in single *K13* genotypes over time might also reflect a better description of local diversity by the wider geographical sampling achieved in 2016.

In villages that reported at least one *P falciparum* case, 49% (290/598) reported their last case (ie, followed by at least 6 months of follow-up by April, 2017) after a median 9 months (IQR 4–14) of malaria post activity. Likewise, 38% (32/85) of village tracts ever presenting *P falciparum* cases reached their last case after a median 13 months (6–19).

The delay from malaria post opening to the last reported clinical *P falciparum* case was 12 months or less for 12% (4/33) of hotspots without mass drug administration, 19% (6/31) of hotspots treated with mass drug administration, and 40% (181/455) of non-hotspot villages. For a delay of 24 months or fewer, these proportions were 33% (7/21), 55% (16/29), and 67% (259/384) respectively. By April, 2017, 965 villages (79%) of 1222 villages corresponding to 104 village tracts were free of *P falciparum* malaria cases for at least 6 months. There was no change in reported malaria-case fatality rate compared with previous estimates (appendix).^23^

## Discussion

Our intensive subnational malaria elimination programme was scaled up to an area encompassing 18 002 km.^2^, ^19^ The key element of the programme was the setup and operation of more than 1200 malaria posts providing early diagnosis and effective treatment, supplemented by mass drug administration in 50 malaria hotspots.^19^ Over 3 years, the incidence of *P falciparum* malaria decreased between 60 and 98% in the different townships, reaching fewer than one case per 1000 individuals per year in three out of the four townships. In hotspot villages, setting up malaria posts alone did not achieve a consistent decrease in incidence. Eliminating the parasite reservoirs by mass drug administration substantially decreased *P falciparum* infections (sustained when verified after 1 year) and clinical *P falciparum* malaria incidence (sustained for >20 months in most hotspots, follow-up still ongoing). This was not associated with worsening drug resistance despite intense drug deployment. The proportion of *K13* wild type parasites remained stable and no piperaquine resistance was detected. The prevalence of *Pfmdr1* multiple copy numbers was 2% in 2016, compared with 60% in this region in 2013, when artesunate-mefloquine was the first-line treatment for *P falciparum* malaria.^22^

There were several strengths and limitations to this study. The programme was largely managed and implemented by members of affected communities through partnership with local community-based health organisations. This partnership facilitated political and logistical support and community acceptance of general interventions (malaria posts) and specific interventions (surveys and mass drug administration). Local and central supervisors monitored activities closely to ensure quality was maintained, data were collected, and supply chains were uninterrupted. Malaria posts were the only access to early diagnosis and treatment in most communities, and therefore provided an accurate representation of malaria incidence and trends. The rollout of the programme took time and careful planning which resulted in the first limitation, a variable duration of follow-up between villages.

Second, only 22% of villages were surveyed to determine the prevalence of malaria infection and hotspot status. It is possible that reservoirs remained undetected. Incidence trends suggest that misclassification is likely to have been minor and contributed only to underestimating the effects of malaria posts in non-hotspots. There were no significant differences in the effects of the malaria post in villages confirmed as non-hotspots and non-surveyed villages when included separately in the model (appendix).

Third, surveys to identify hotspot villages used ultrasensitive PCR to detect and quantify malaria infection. Although a high-throughput method was developed for this project, wider rollout of this method would require considerable investment. Alternative methods for identification of hotspots would be desirable.^24^, ^25^

Fourth, no direct randomised comparisons were done between mass drug administration and control hotspots. As *P falciparum* infection prevalence and clinical incidence clustered, to leave hotspots untreated as controls would have been unethical and likely to fuel reinfection of intervention villages. Hotspots therefore received mass drug administration as soon as possible after identification. The absence of follow-up surveys in non-hotspot villages prevented us from excluding formally that our findings were liable to a degree of regression bias.^26^ Some villages with high prevalence may have returned to levels similar to other villages without intervention. Considering that *P falciparum* carriage is influenced by complex social, economical, entomological, meteorological, and epidemiological forces and that these forces do not necessarily vary from year to year (eg, the spatial clustering of prevalence in spite of different seasons or years of surveys), the contribution of regression bias to the findings is likely to be small.

Vector control for malaria is less effective in the Greater Mekong subregion than in other endemic regions and malaria control is therefore disproportionately dependent on the use of antimalarial drugs.^8^, ^9^ *P falciparum* parasites in this region have developed resistance to artemisinin, its derivatives and most of its partner drugs, leading to rising rates of treatment failure. The emergence and spread of resistant malaria parasites could therefore undermine all elimination efforts in the Greater Mekong subregion. If these resistant parasites spread to high burden countries in south Asia and Africa, as resistant parasites have done in the past,^27^, ^28^ a humanitarian disaster could result.^29^ Given that there are no readily available alternative antimalarials, and that resistance is both increasing and spreading, rapid elimination of *P falciparum* infection might become the only viable, timely solution.

This study confirms the key importance of access to timely, appropriate case management,^6^ and the contribution of targeted mass drug administration in a few villages with high prevalence of *Plasmodium* infection.^17^, ^30^ The success of mass drug administration was achieved by consistent high participation in mass drug administration activities, obtained from an intensive community engagement work at all stages, from planning interventions during seasons compatible with the agricultural calendar, to rumour control during delivery.^31^ Additionally, access to treatment was also probably crucial in preventing the replenishment of the reservoir after mass drug administration, particularly to limit the effect of population movements. This study shows that *P falciparum* can be safely and rapidly decreased to zero (at least for the duration of the observation period) from populations residing in large areas that are difficult to access—an achievement hitherto often considered impossible. If elimination activities can proceed in all contiguous endemic areas in addition to the standard control programmes already implemented,^32^ and gains are secured through sustained access to community-based early diagnosis and treatment, epidemiological surveillance, and rapid response, there is a real possibility of subnational elimination of *P falciparum* before drug resistance reverses all these gains. This outcome will require immediate and continued commitment and adequate funding.
