# Supplementary material for: Effect of generalised access to early diagnosis and treatment and targeted mass drug administration on Plasmodium falciparum malaria in Eastern Myanmar: an observational study of a regional elimination programme
Source: Lancet. 2018 May 12;391(10133):1916–26. doi: 10.1016/S0140-6736(18)30792-X (PMC5946089; doi:10.1016/S0140-6736(18)30792-X)
Supplement: Supplementary appendix [file mmc1.pdf]

THE LANCET

Supplementary appendix

This appendix formed part of the original submission and has been peer reviewed. We post it as supplied by the authors.

Supplement to: Landier J, Parker DM, Myint Thu A, et al. Effect of generalised access to early diagnosis and treatment and targeted mass drug administration on *Plasmodium falciparum* malaria in Eastern Myanmar: an observational study of a regional elimination programme. *Lancet* 2018; published online April 24. http://dx.doi.org/10.1016/S0140-6736(18)30792-X.

APPENDIX: Effect of generalised access to early diagnosis and treatment and targeted mass drug administration on Plasmodium falciparum malaria in Eastern Myanmar: an observational study of a regional elimination programme.

Jordi Landier, Daniel M. Parker, Aung Myint Thu, Khin Maung Lwin, Gilles Delmas, François H. Nosten, for the Malaria Elimination Task Force Group.

PROGRAM DEPLOYMENT

The deployment of the malaria posts (MP) network was gradual, following a rough east to west spread (Figure 1 and 2). Since July 2016, the Malaria Elimination Task Force (METF) MP network extended to 1,222 villages in 157 village tracts, and covered 82% of villages mapped in the target area (1,222/1,490), providing access to malaria EDT to an estimated 365,000 rural villagers (MPs were not established in urban areas). The remaining 18% uncovered villages were either already included in a previous program of a partner organization (mainly in Kawkareik and Hlaingbwe townships), close enough from an existing MP to rely on its services (<1km) or where the number of households was low and no suitable MPW could be found.

Villages were matched by coordinates to administrative units of the national Myanmar system using data available from the Myanmar Information Management Unit (http://themimu.info/). This system includes (in order from largest to smallest): States/Regions, Districts, Townships, and Village Tracts. Village population size was calculated as *number of households collected during mapping*average household size* obtained from villages where a complete census was obtained during specific activities (e.g. MDA, survey). The median number of villages per village tract was 6 (Interquartile range (IQR)=3-10, n=157)), corresponding to a median of 2,650 inhabitants per village tract (IQR=1,245-4,450). The median village population was 260 inhabitants (IQR=140-480, n=1,222). Of note, the northernmost township (Hpapun) had larger village counts per village tract (median=13, IQR=7-21, n=36), and smaller villages (median=140, IQR=100-220, n=478).

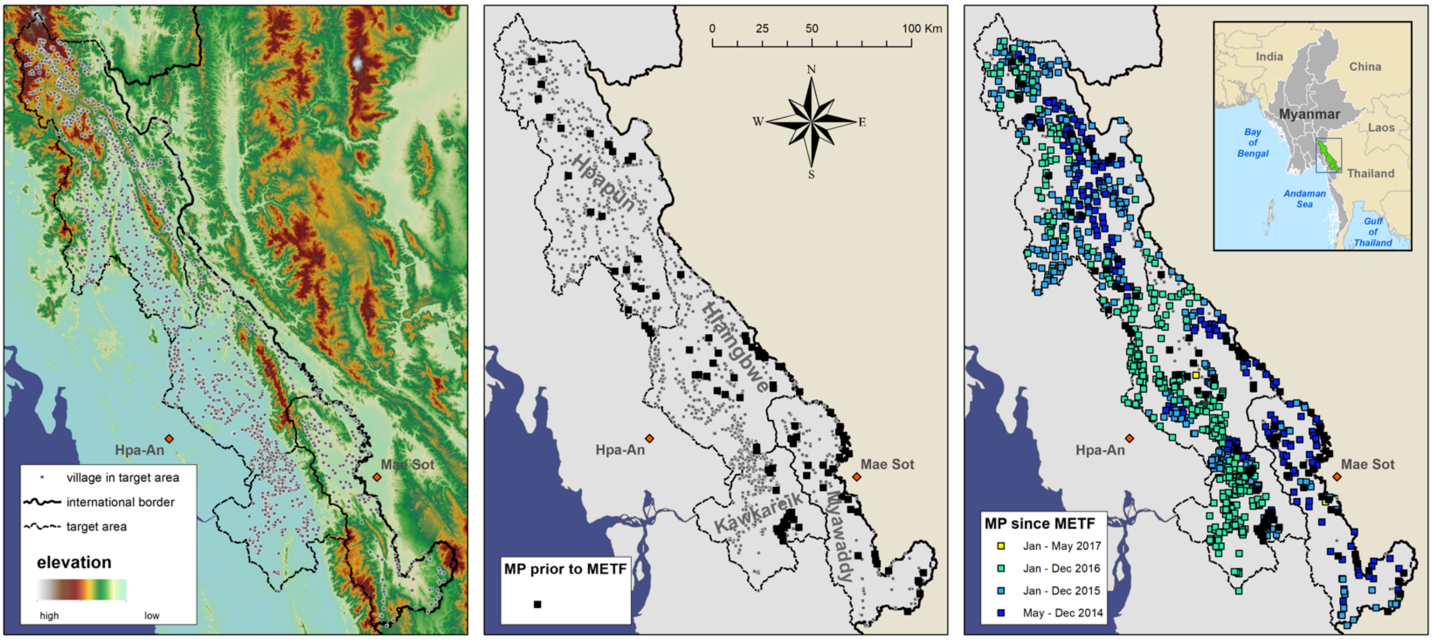

Figure S1: A: Physical geography of the METF target region; B: Access points to malaria EDT before

the start of the programme in April 2014 (n=113); C: METF Malaria Post (MP) network as of April 2017 (n = 1,222).

Malaria Posts (MP)

Between 1 May 2014 and 30 April 2017, 633,771 person.years of follow-up were recorded, 238,682 fever cases were seen by MP workers, 240,362 RDTs were used for malaria diagnosis, and 10,791 *P. falciparum* and 17,490 *P. vivax* cases were diagnosed and treated. A total of 52 severe malaria cases and 8 malaria-related deaths were reported by MP. Assuming that *P. falciparum* was the cause of these severe cases and deaths, severe cases represented 0.5% of 10,791 cases (95%CI=0.4-0.6) with a case-fatality rate of 0.7 per 1000 (95%CI=0.3-1.5) [18]. In 80% (161,694/201,114) of fever cases with individual data, MP were accessed within 48h of fever onset, corresponding to consultation after an average delay of 1.8 days since fever onset (median=1 day, IQR=1-2). A total of 107,497 weekly reports were analyzed. Missing reports were limited: 73% (898/1222) of villages presented no missing report, 15% (178/1222) had a single missing report, 5% (64/1222) had 2 missing reports, and 7% (82/1222) had 3 or more missing reports. In 11 villages, 10 to 46 weekly reports were missing as a result from interrupted MP services. In 6 villages, the MP activity stopped permanently after 10 to 85 weeks. The main reason for permanent interruption of services was armed conflict which made MP worker activity impossible. One hotspot became inaccessible for further intervention (MP set-up or MDA) because of armed conflict that occurred after the survey.

Surveys

A total of 272 baseline prevalence surveys were conducted between April 2014 and January 2017, which identified 69 hotspots. The median surveyed population for surveyed villages was 207 inhabitants (IQR=122-327). The median number of samples collected per village was 47 (IQR=37-54), which corresponded to a median 21% of the village population included in the survey (IQR=13-32%), and assuming 50% of adults in the village population, a median 42% (IQR=27-64%) of adult population included in the survey. In 10 villages, the number of samples collected was too low and the prevalence was not estimated with sufficient precision. Between January 2016 and April 2017, 42 villages had reached 12 months after MDA campaign and 40 were surveyed again. For M12 surveys, the median number of samples collected per village was 70 (IQR=50-109). This corresponded to a median of 39% (IQR=32-45%) of village population included in the survey, or, assuming 50% of adults, a median 78% (IQR=65-91%) of adult population included.

Hotspot definition

The survey samples were analysed by ultrasensitive qPCR which detects parasitemia as low as 20 parasites/mL of blood, circa 75% of the total parasite reservoir (Imwong et al, Journal of Infectious Diseases 2016). This differs significantly from usual prevalence surveys using RDT or microscope. In the GMS, the sensitivity of these methods is close to the patency parasitemia threshold. Here, the prevalence values that are obtained from these surveys also estimate the size of the sub-microscopic reservoir of parasites.

In the absence of pre-established threshold to define a hotspot, especially using ultrasensitive qPCR as a detection method, the hotspot definition was chosen to be coherent with that used in the pilot study (see Landier et al, Wellcome Open Research 2017) and according to a pragmatic risk-benefit assessment: the lower the threshold, the more MDA campaigns would have to be conducted,

increasing the proportion of parasite-free individuals that would be treated. This definition limited the MDA campaigns to 5% of the villages with the highest parasite burden.

MDA delivery and safety

MDA was conducted in 50 hotspot villages in 5 campaigns between January 2015 and December 2016. The median duration of MP activity before MDA was 12 months (IQR=5-16, n=50), and the median duration between baseline survey and MDA was 5 months (IQR=4-6, n=50).

Participant age and sex, MDA participation and reasons for not participating were recorded. A population list provided in advance by the village headman allowed to document absentees. This data was used to calculate the proportion of the population participating to a given number of MDA rounds of treatment, as: *number of persons taking 0, 1, 2, or 3 rounds / total number of village inhabitants present in the village at least once during the 3 months of intervention*.

The total target population for MDA was 12,465 persons. Of these, 7,444 (60%) took 3 consecutive rounds of treatment; 2,000 (16%) took 2 rounds; 1,601 (13%) took 1 round; and 1,420 (11%) were not treated. The proportion (median, n=50 villages) of the population taking all 3 rounds was 64% (IQR=50-78%) and taking at least 1 round was 91% (IQR=86-95). Over the 3 months the 12,465 individuals were met a total of 33,317 times to begin a round of treatment. Treatment was initiated on 28,353 (86%) occasions and refused on 3,593 (11%) occasions, while eligibility criteria were not met on 1,371 (4%) occasions. Delivery of the intervention was generally successful, with 98.5% (27,936/28,353) of initiated treatments that completed and 97.6% (82,346/84,401) of doses taken under supervision. Detailed adverse events records were analyzed for 15 villages. A total of 132 (1.5%) adverse events were reported out of 8,774 treatments in 15 villages with DP and PQ. The most commonly reported symptoms were dizziness 26%, headache 16%, nausea 12%, itchiness 6%, anorexia 8%, gastrointestinal symptoms 11%, heart palpitation 5%, sleep problems 11%, and fatigue 4%. There were no reports of serious adverse reactions.

The median duration of MP activity in hotspots after MDA and until 30 April 2017 was 20 months (IQR=14-24, n=50). MDA in the 18 remaining accessible hotspot villages was scheduled between July and December 2017.

DESCRIPTIVE RESULTS

Temporal trends in malaria incidence in the four main townships targeted by METF

The magnitude of the seasonal incidence peaks (high-transmission periods in June and December) in Hpapun township decreased, as well as the overall incidence between peaks. Seasonal *P. falciparum* incidence peaks ceased in Hlaingbwe, Myawaddy and Kawkareik townships after 1 year.

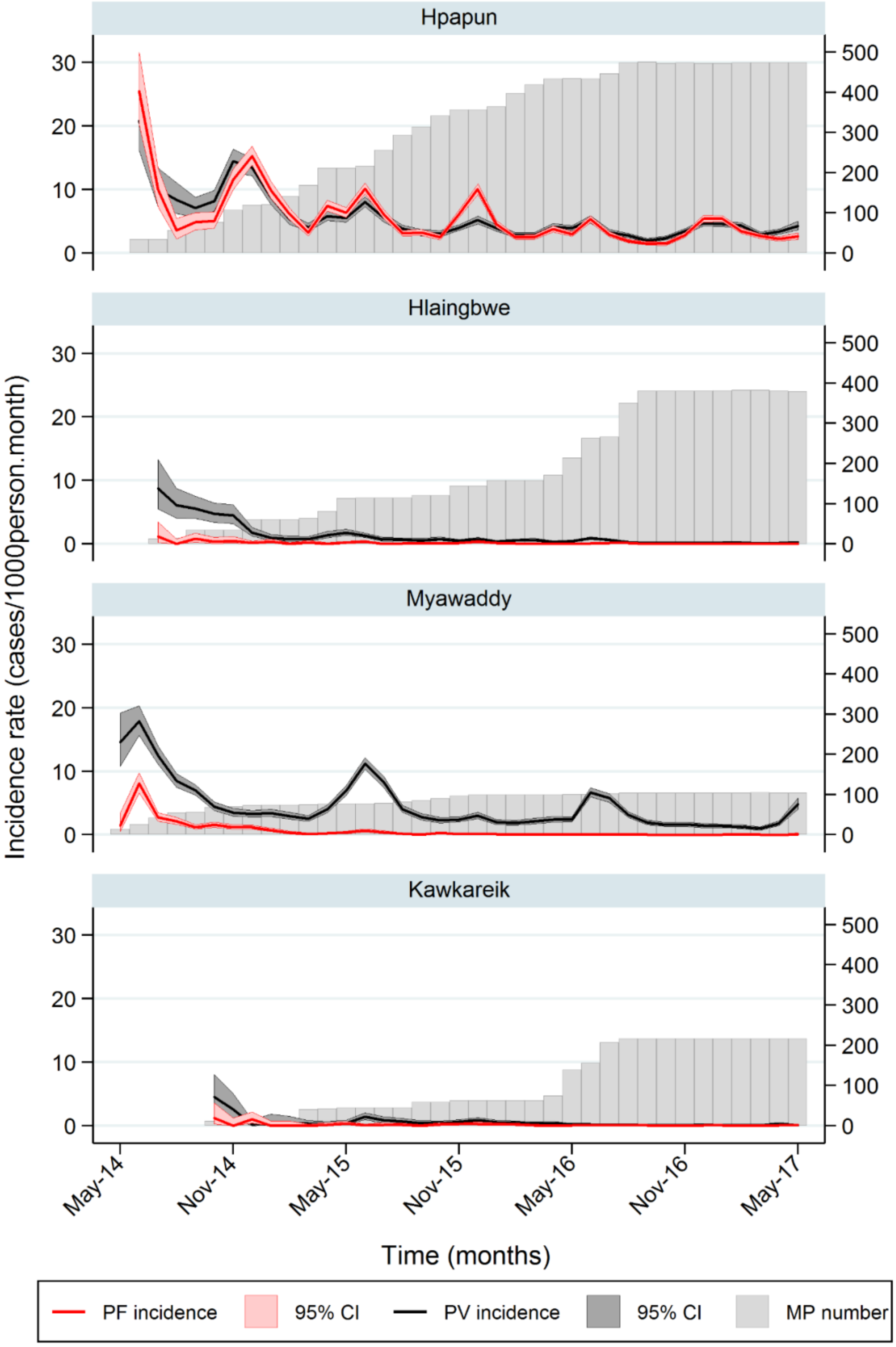

Figure S2: Incidence of clinical malaria episodes at the township level over 3 years (May 2014-April 2017).

Mortality

A total of 52 severe malaria cases and 8 malaria-related deaths were reported by MP. Assuming that *P. falciparum* was the cause of these severe cases and deaths, severe cases represented 0.5% of 10,791 cases (95%CI=0.4-0.6) with a case-fatality rate of 0.7 per 1000 (95%CI=0.3-1.5).

Detection of falciparum and vivax infection by ultrasensitive qPCR

In 272 baseline surveys including 12,110 samples, a total of 2,669 malaria infections were detected by qPCR, corresponding to 551 *P. falciparum* (4.6%), 1,453 *P. vivax* (12.0%), 196 mixed (1.6%), and 469 (3.9%) Plasmodium spp (undetermined species) infections. In 40 M12 surveys, a total of 767 malaria infections were detected by qPCR on 3,288 samples, corresponding to 59 *P. falciparum* (1.8%), 576 *P. vivax* (17.5%), 13 mixed (0.4%), and 119 (3.6%) Plasmodium spp (undetermined species) infections.

Non-falciparum, non-vivax Plasmodium infections represented 2.2% (11/507) of microscopy positive with 9 *P. malariae*, 1 *P. ovale* and 1 *P. falciparum* and *P. malariae* mixed infection.

Table S1: Proportion of malaria infections detected by microscopy and RDT for 3388 samples found positive by ultrasensitive qPCR and analysed with all three methods, showing the large submicroscopic, sub-RDT reservoir in this setting.

	qPCR	microscopy	RDT
P. falciparum (PF)	603	164 (27%)	201 (33%)
P. vivax (PV)	2003	231 (12%)	76 (4%)
P. falciparum+other species (PF+PV or PF+PM)	206	81 (39%)*<br>1 PF+PM<br>51 PF<br>10 PF+PV<br>19 PV	72 (35%)<br>68 PF<br>1 PF+PV<br>2 PV
Plasmodium spp	576	14 (2%)‡	7 (1%)
P. malariae (PM)	Not tested	10	Not tested
P. ovale (PO)	Not tested	1	Not tested

* including 1 *P. falciparum+P. malariae* mixed infection ‡including 7 *P. malariae* infections

Spatial distribution of incidence and prevalence

The vast majority of high prevalence villages were located in the northernmost area (Hpapun Township), where incidence was also highest (**Figure S2 and S3**). Out of 210 villages that were randomly selected for malaria surveys 35 were identified as malaria hotspots using the operational definition. 60% of these hotspots (21/35) were within 5km of another hotspot that had been identified through random selection and 94% (33/35) were within 10km of another randomly selected hotspot (**Figure S4**).

The median distance between randomly selected non-hotspot villages and their nearest hotspot neighbors was 4.27 times further than the distances between hotspot villages and their nearest hotspot neighbors (median: 18.17/4.26; W = 4997; p-value = 0.0001).

Spatial autocorrelation was evident from maps of village prevalence and from several measures of spatial autocorrelation (**Figures S2, S3, S4**). The vast majority of high *P. falciparum* prevalence villages were in the northernmost township of the target area (Hpapun Township). Clustering of village prevalence was strongest among nearest neighbors and decreased until clustering was no longer evident at villages that were 60 - 70km away from each other (**Figure S5**). At a lag of 75km there was a negative correlation, indicating that villages at this distance from each other in the target area have very different prevalences. A similar pattern existed for incidence of *P. falciparum* malaria (**Figure S6**).

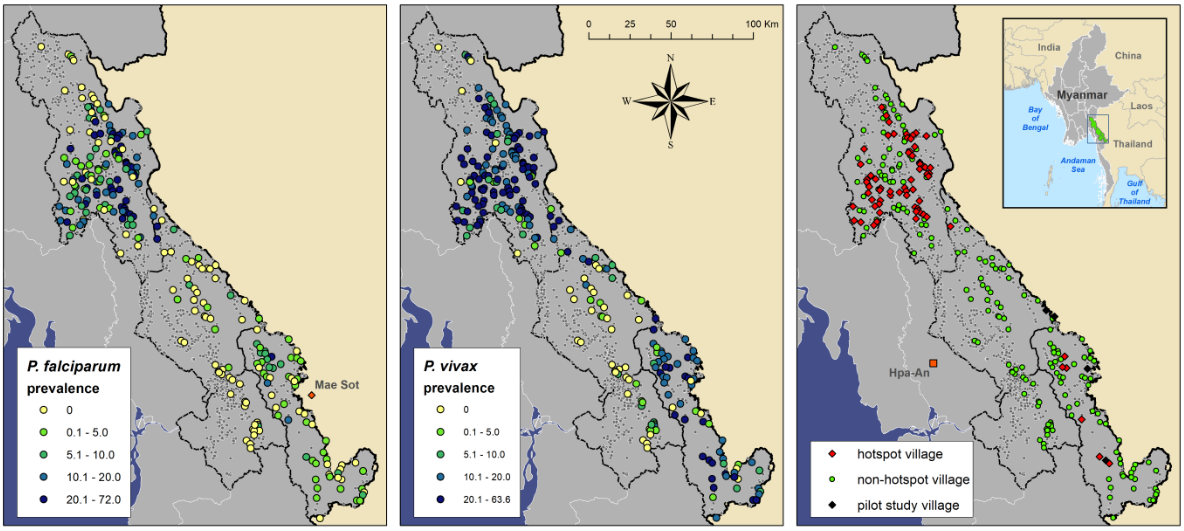

<u>Figure S3: Location of surveyed villages showing the spatial distribution of asymptomatic infection prevalence for *P. falciparum* (left panel) and *P. vivax* (central panel), as well as the spatial distribution of hotspot villages (right panel).</u> Hotspots were mainly found in the northernmost division (Hpapun Township), where incidence was also highest (Figure S2). Hotspots also clustered at the local scale.

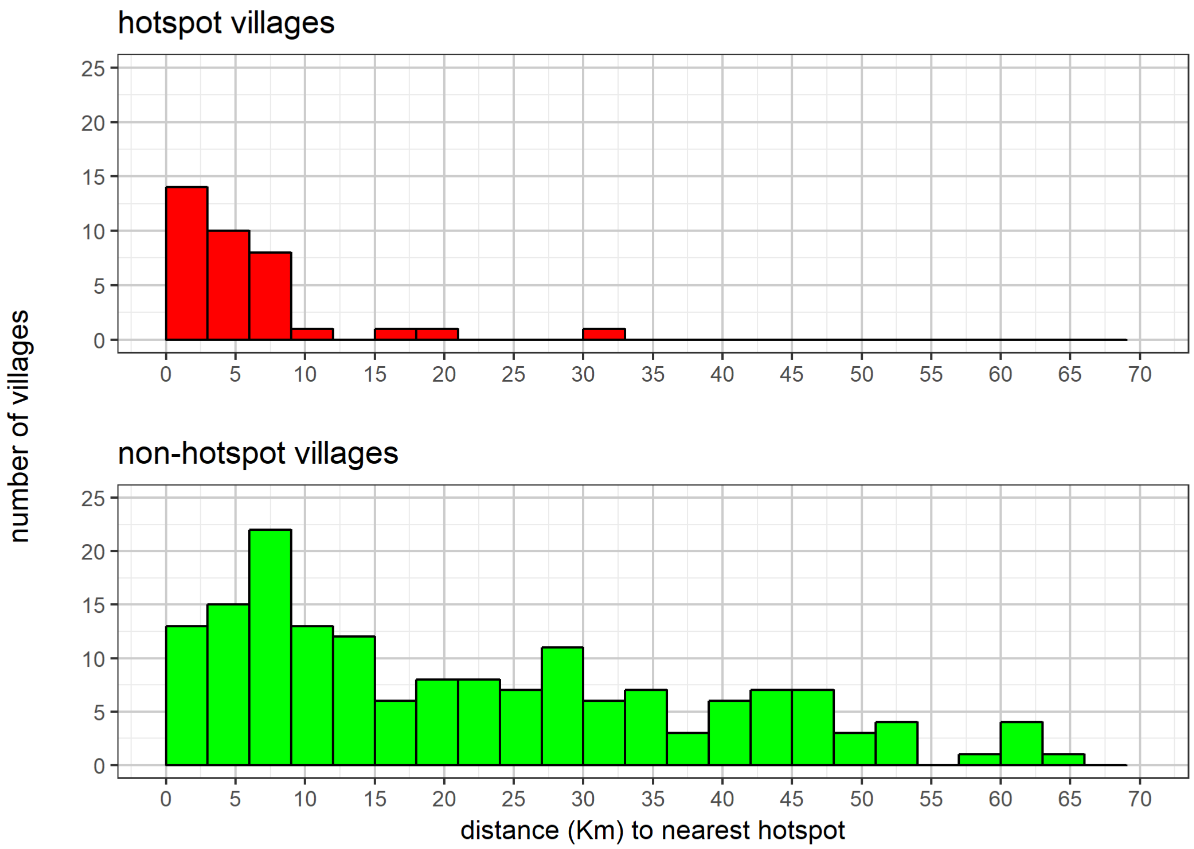

Figure S4: distribution of the distances between villages and their nearest hotspot neighour: Hotspot villages (from the operational definition, with 40% malaria, of which 20% is Pf) and their nearest hotspot neighbors (red) and non-hotspot villages and their nearest hotspot neighbors (green). Hotspot villages were usually tightly clustered near other hotspot villages.

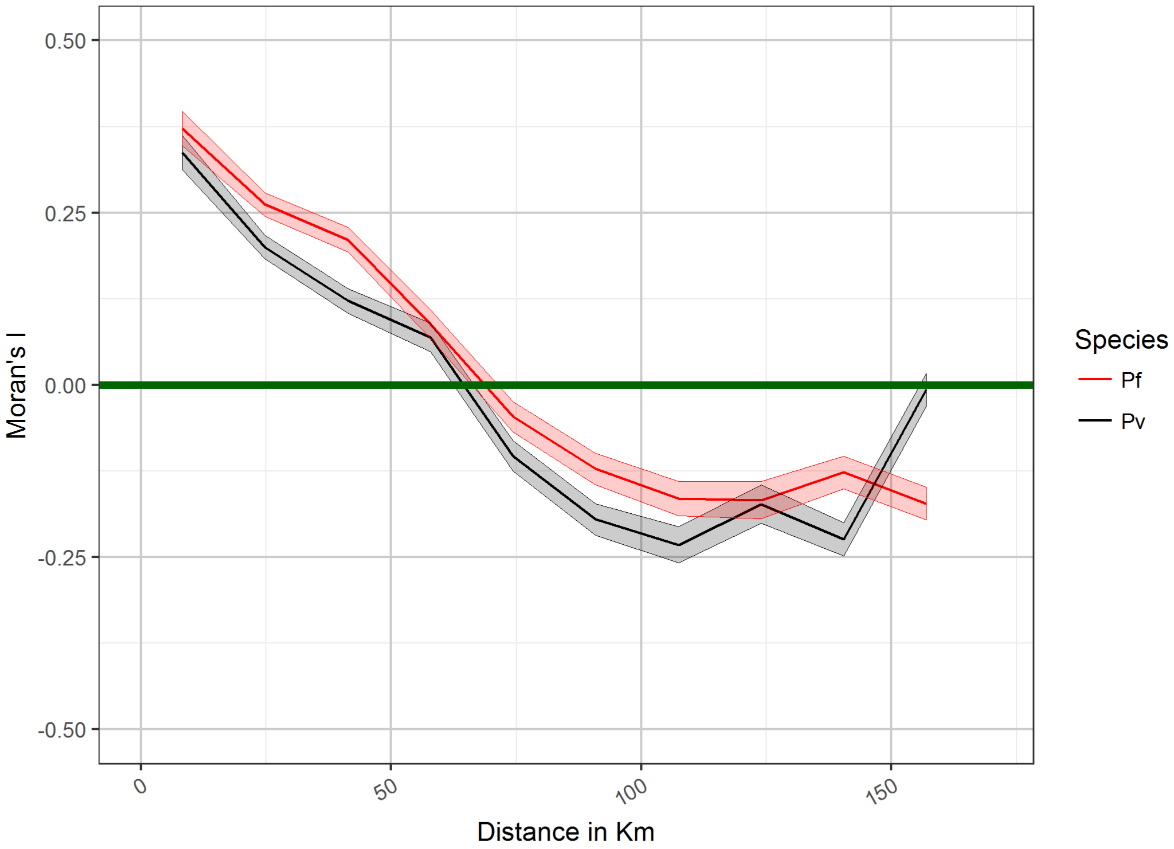

<u>Figure S5: Spatial correlogram indicating clustering of villages by prevalence for both *P. falciparum* (red) and *P. vivax* (black).</u> The Moran's *I* statistic on the y-axis indicates the magnitude of autocorrelation between villages for a given distance band (on the x-axis). High clustering is positive and increasing as it approaches 1 whereas negative clustering (dispersal) is indicated by negative values approaching -1. Villages in close proximity exhibit similar levels of prevalence (high or low), regardless of the species. These clusters are strongly dependent on geographic proximity. Clustering is absent at villages that are 60km apart while at distances of 75 to 100km there is evidence of strong negative correlation.

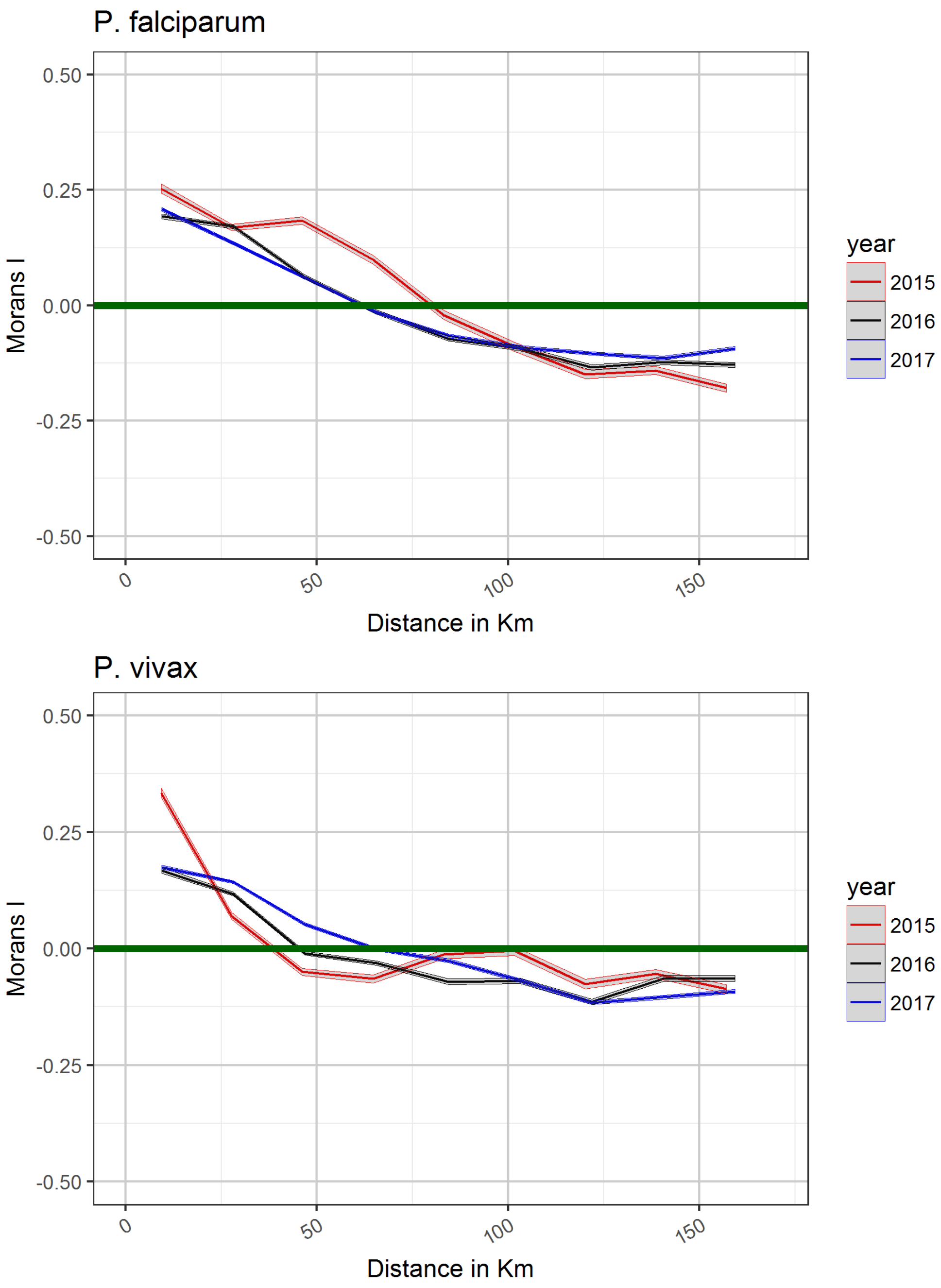

<u>Figure S6: Spatial correlogram indicating clustering of villages by incidence for both *P. falciparum* (top panel) and *P. vivax* (bottom panel) over the three years of the project:</u> During year one of the project spatial clustering was apparent even at distances larger than 50 km while in 2016 and 2017 the clustering had decreased to smaller geographic areas.

Seasonality and malaria infection prevalence by ultrasensitive qPCR

Seasonal malaria incidence appeared to have a small influence on underlying prevalence levels as detected through qPCR. In Hpapun Township the median prevalence measured by uPCR across 143 villages was 9% (IQR=3-18%) for *P. falciparum* and 21% (IQR=12-31%) for *P. vivax*. Only minimal differences were observed between prevalence of either species in surveys conducted in February-May (low transmission), June-July (high transmission), in August-October (low transmission), or in November-January (high transmission) and the differences were not statistically significant (p=0.15 for *P. falciparum*, p=0.32 for *P. vivax*, Kruskall-Wallis test). Since there was no longitudinal follow-up of villages in the absence of intervention this represents only indirect evidence. However, the relative stability in the distribution of prevalence across transmission periods seems to exclude deep seasonal oscillations, suggesting that seasonal fluctuations are unlikely to explain the differences observed between *P. falciparum* prevalence at baseline and 12 months post MDA (Figure S7A).

P. falciparum prevalence estimates above 40% (n=6 villages) were limited to high transmission months and in 3/6 villages corresponded to an excess of clinical cases due to ongoing high transmission at the time of survey (as expected). Overall this suggests that seasonality-driven transmission exerts a marginal influence on a relatively stable reservoir. This is supported by an unpublished analysis of risk factors for individual level malaria infection, where the probability of infection was strongly associated with yearly cumulative incidence within a village.

IMPACT RESULTS

Impact of MDA on hotspots malaria incidence and prevalence

The decrease in *P. falciparum* incidence after MDA was accompanied by a species-specific reduction of the asymptomatic malaria reservoir. The median decrease in the *P. falciparum* prevalence was 92% (IQR=81;100, n=40) when compared to baseline (p<0.0001 when testing the difference from 0 using the Wilcoxon matched-pairs sign rank test) (Figure S7A). The median decrease in *P. vivax* prevalence was 19% (IQR=47% lower; 8% higher) in the *P. vivax* reservoir (p=0.02) (Figure S7B).

In 41 villages with >20 weeks of follow-up before and after MDA, the median unadjusted incidence of falciparum clinical episodes dropped from 125.0 cases/1000/year (IQR=31.7-195.8) over the 12-month period before MDA to 15.0 (IQR=8.7-39.5) after (Figure S7C). The median change was -79% (IQR=-95; -60). Incidence data up to 24 months before and after MDA exhibited similar trends (Figure S8).

Figure S7: Impact of MDA on malaria in villages equipped with MP and addressed with MDA between January 2015 and April 2016.

A. *P. falciparum* prevalence (95% confidence interval) comparing baseline and 12 months after MDA (n=40 villages);

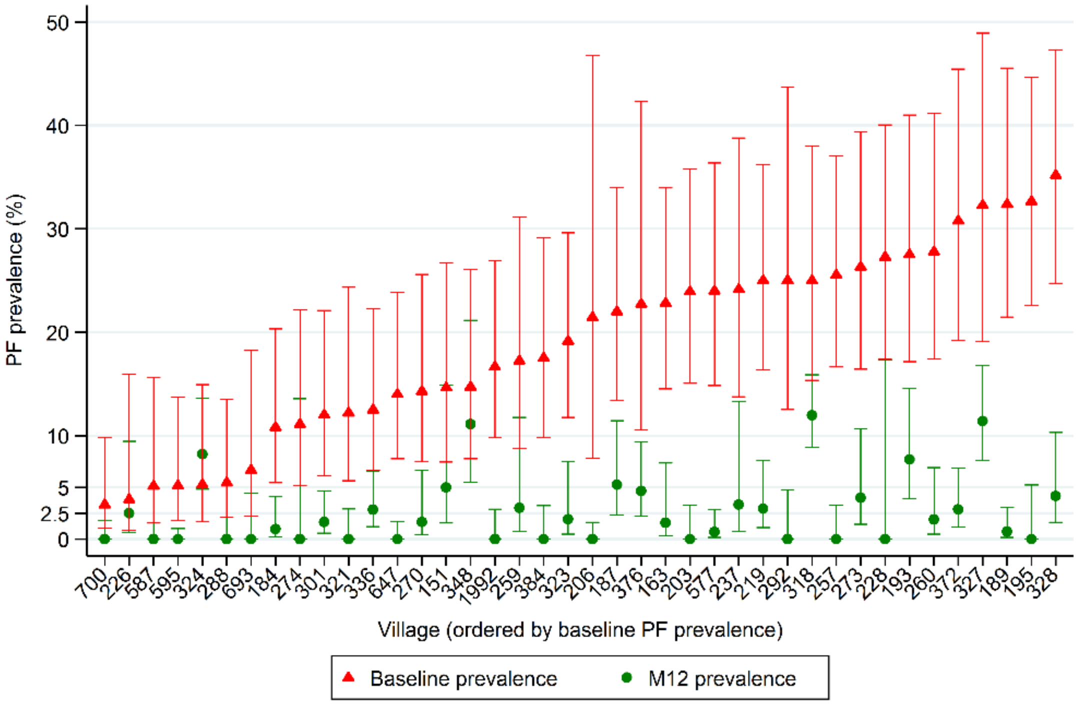

B. *P. vivax* prevalence (95% CI) comparing baseline and 12 months after MDA (n=40);

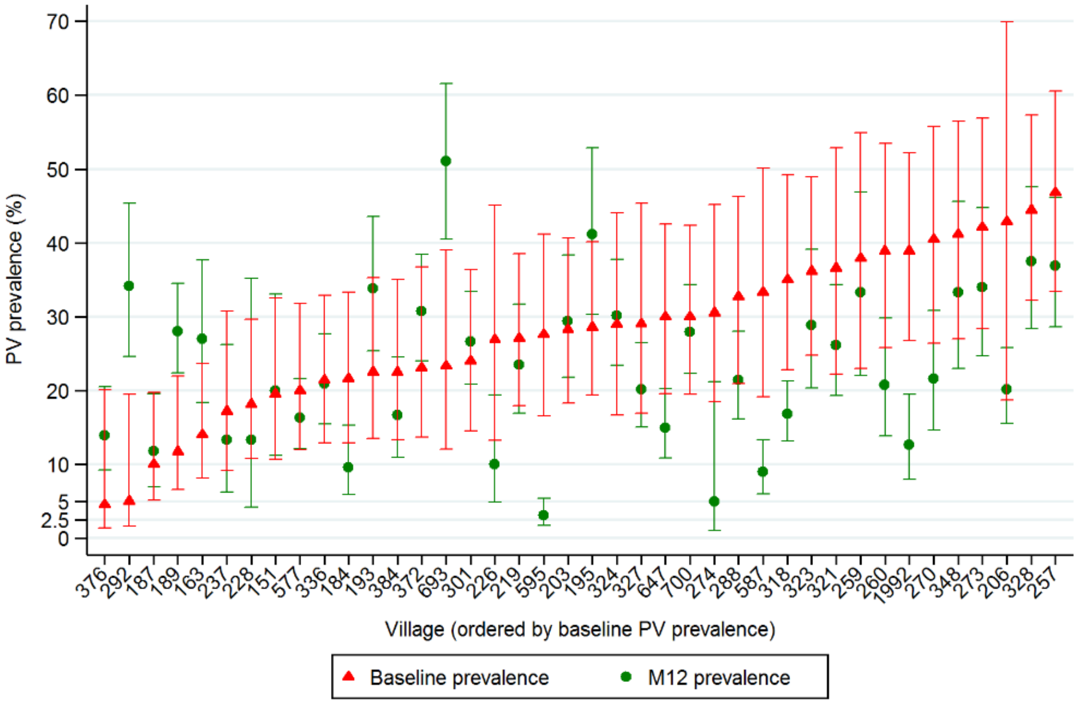

C. Cumulative incidence of clinical *P. falciparum* malaria comparing the year before MDA and the year after MDA. Cumulative incidence before MDA is not shown for 9/68 villages (13%) where MP was opened <20 weeks before MDA and would provide an unreliable cumulative incidence estimate (see Figure S8 for longer follow-up).

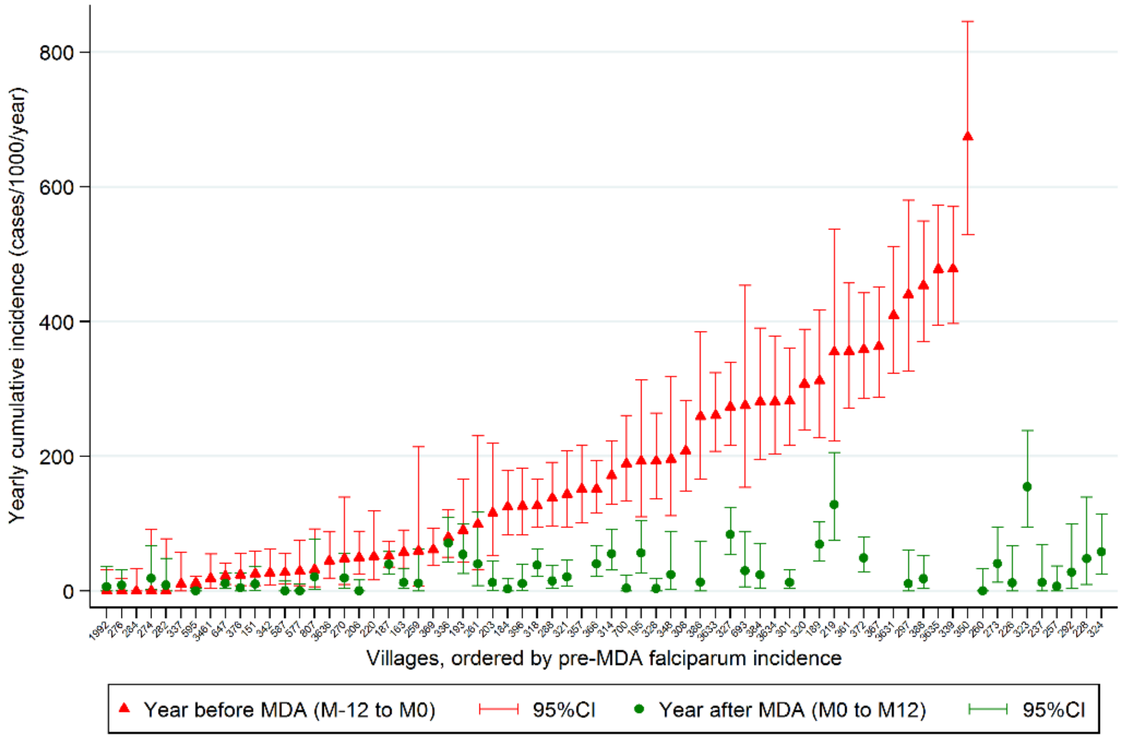

D. Incidence of clinical *P. vivax* malaria. Cumulative incidence before MDA is not shown for 9/68 villages (13%) where MP was opened <20 weeks before MDA and would provide an unreliable cumulative incidence estimate (see Figure S8 for longer follow-up).

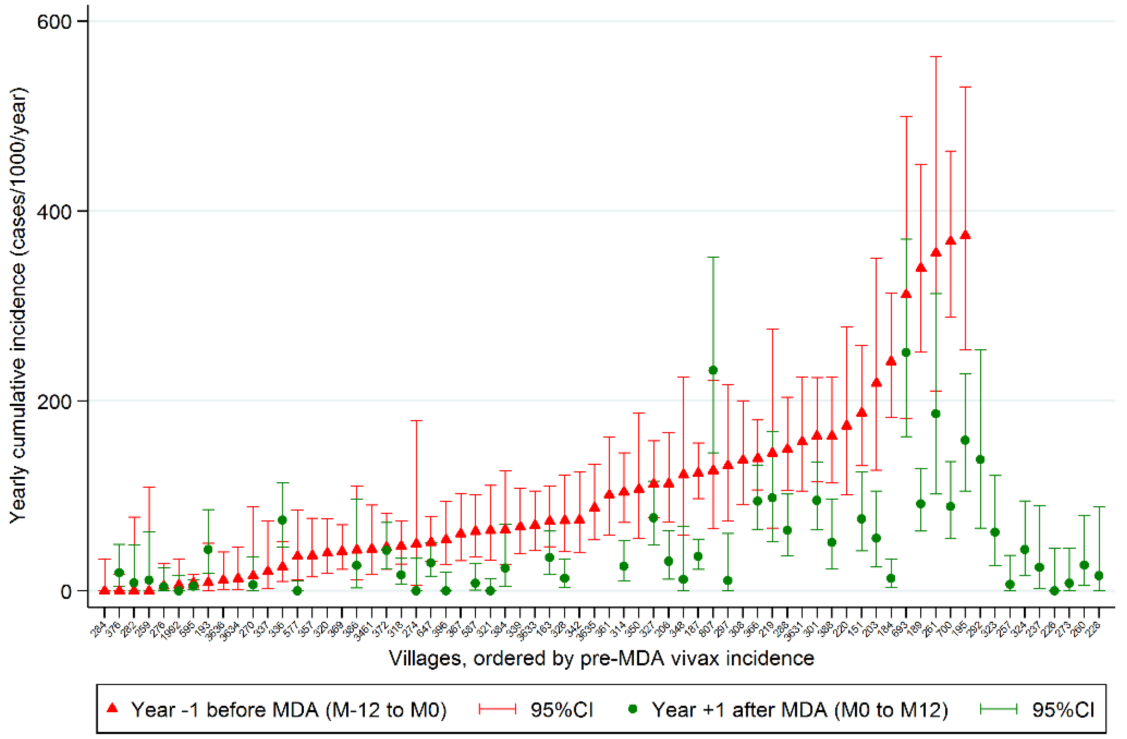

Figure S8: Yearly cumulative incidence of clinical malaria comparing the two years before MDA and the two years after MDA (excluding villages with <20 weeks of follow-up in a given period); extended from Figure S7C and S7D.

A. ***P. falciparum***

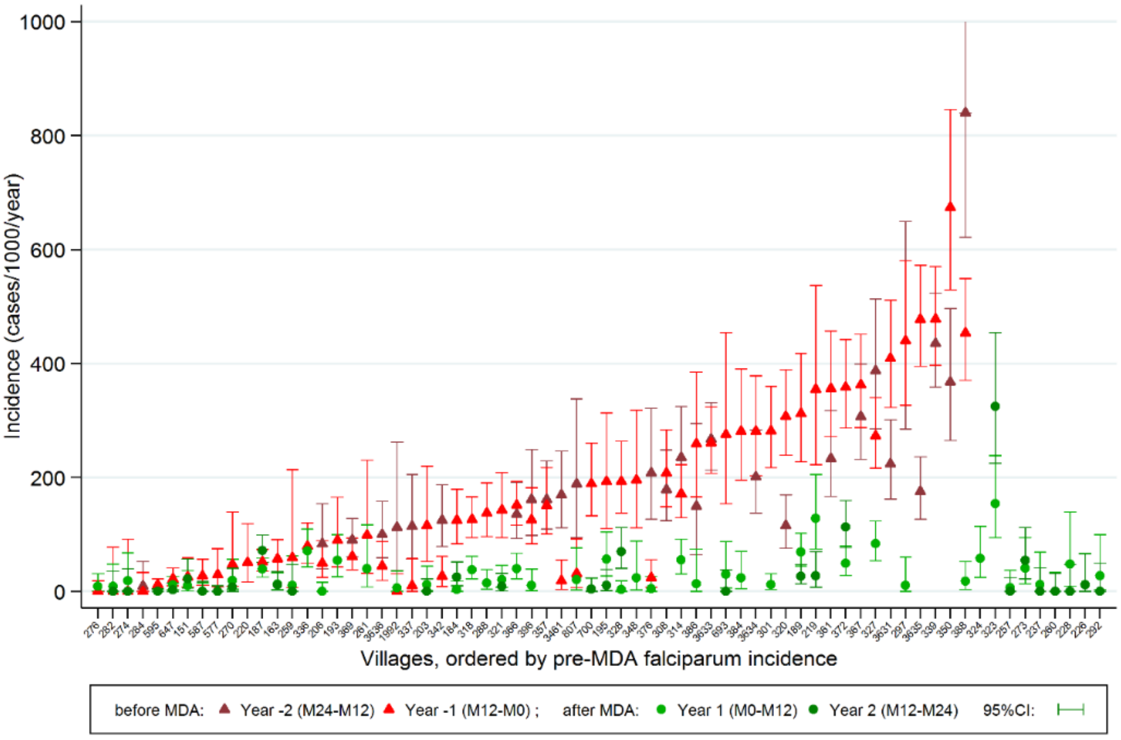

B. ***P. vivax***

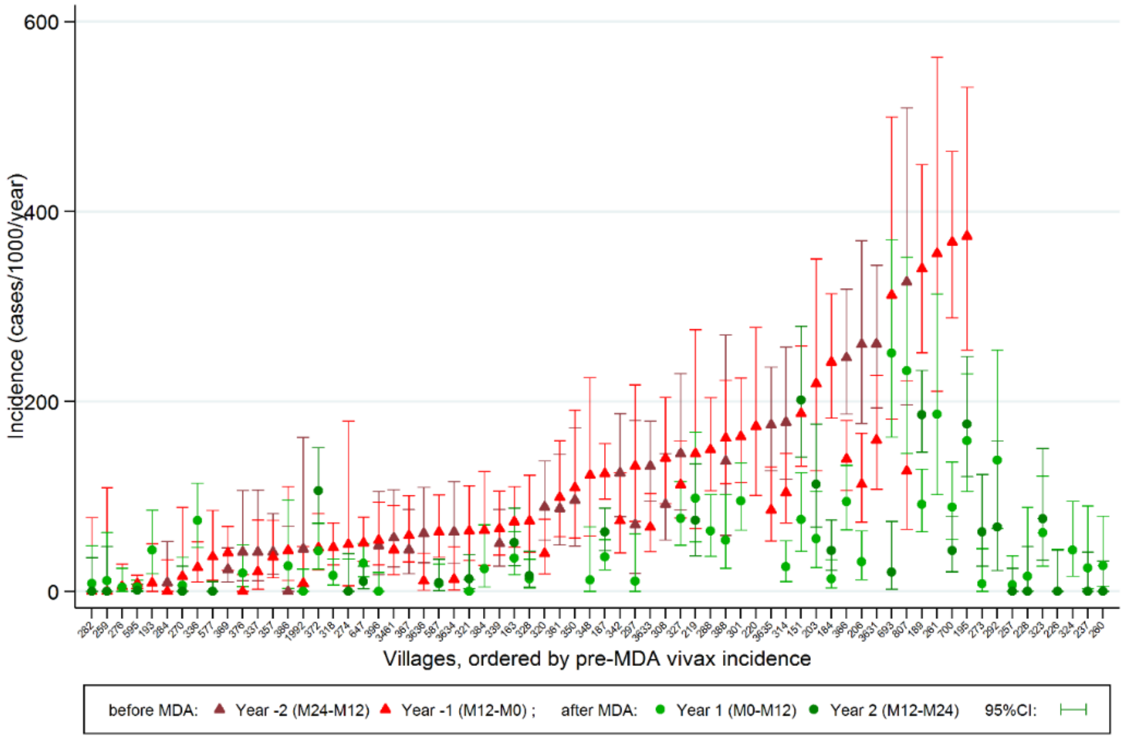

Impact of the program on adjusted malaria incidence

Supplementary methods

The change in village-level *P. falciparum* clinical case incidence over time was analysed in a generalized additive multilevel negative binomial model. The negative binomial model was preferred as it allowed to account for over-dispersion of case counts. Adjustments for spatial and temporal autocorrelation (seasonality, latitude, and longitude, using spline functions; altitude as a categorical variable) were included. Splines were used to quantify and control for the added risk related to geographic location or season without assuming a specific shape in the relationship between these parameters and malaria incidence. Village population size (log-transformed) was included as an offset and random effects (for both intercept and slope) at the village level were used to account for unexplained local heterogeneity. The contribution of the duration of MP activity in a village, MDA intervention, and the proportion of villages within the same village tract equipped with MPs were estimated.

The duration of MP activity in a village was modelled as a linear function of time and the validity of the linear assumption was explored using spline functions. When the linear assumption was not verified, other specifications of the duration of MP activity were tested based on the shape of the spline. An interaction between duration of MP activity and the village and the type of village (non-hotspot, hotspot before MDA, hotspot after MDA) was included and tested. The impact on *P. vivax* was evaluated using the same model and set of variables.

Table S2: Univariate analysis (without latitude, longitude, and season adjustment).

Variable		*P. falciparum*			*P. vivax*		
		IRR	95%CI	p-value	IRR	95%CI	p-value
MDA status	No MDA (Non-hotspot)	1	reference	<0.0001	1	reference	
	Hotspot before MDA	69.1	36.5-131		19.04	11.1-32.6	
	Hotspot after MDA	20.9	10.8-40.4		6.9	4.0-12.2	<0.0001
Duration of MP activity in the village (linear approximation, per 1 quarter increment)		0.71	0.69-0.73	<0.0001	0.90	0.88-0.92	<0.0001
MDA status x duration of MP activity (per 1 quarter increment)	No MDA (Non-hotspot)	1	reference	<0.0001	1	reference	<0.0001
	Hotspot before MDA	57.4	32.5-101.1		20.6	12.1-34.8	
	Hotspot after MDA	5.4	2.9-10.1		3.9	2.2-6.9	
	Duration in No MDA	0.70	0.68-0.72		0.90	0.88-0.92	
	Duration in Hotspot before MDA	0.76	0.70-0.82		0.89	0.84-0.95	
	Duration in Hotspot after MDA	1.01	0.94-1.10		1.06	1.00-1.12	
MP coverage of villages within the village tract (per 10% coverage increment)		0.71	0.69-0.73	<0.0001	0.78	0.76-0.80	<0.0001
Number of MDA conducted within the village tract excluding the village (per 1 village increment)		0.80	0.77-0.83	<0.0001	0.90	0.87-0.93	<0.0001
MP embedded in a community clinic		2.06	1.02-4.16	0.044	3.5	2.0-6.0	<0.0001
Altitude	0-40m	1	reference	<0.0001	1	reference	<0.0001
	40-250m	26.7	17.0-42.0		13.3	9.5-18.6	
	250m-500m	66.5	39.4-112.4		33. 5	22.5-49.8	
	500m-900m	43.4	24.1-78.2		31.0	19.9-48.3	
	900m-1500m	6.0	2.9-12.6		5.9	3.5-10.1	
	missing	2.5	0.4-16.3		2.0	0.5-8.0	

Altitude, latitude and longitude were closely related (see also Figure 1). The adjustments for location and season are presented in Figure S9. After adjusting for latitude and longitude, a small risk increase persisted at an altitude of 250-500m (IRR=1.7; 95%CI=1.1-2.5 for *P. falciparum* and IRR=1.5; 95%CI=1.1-2.0 for *P. vivax*) compared to the lowest altitude category (0-40m). No other altitude category between 40m and 250m and $\geq$500m) was associated with an increased risk.

P. falciparum seasonality presented the two characteristic peaks: one narrow peak at the onset of rainy season, between week 21 (June) and 29 (mid-July), and one larger peak during the cold season between week 46 (mid-November) to week 5 (end of January). *P. vivax* seasonality was strongly associated with a peak at the beginning of the rainy season, between week 19 (mid-May) and 31 (end of July).

The baseline adjusted-risk map at MP opening provided by the latitude/longitude splines shows a high increase in risk for *P. falciparum* in Hpapun township, limited in the north by higher altitude (>1000m). Malaria risk (both *P. falciparum* and *P. vivax*) was lowest in the western parts of Hlaingbwe and Kawkareik townships, corresponding to low altitude agricultural plains with little forest cover or mountains. In the central and southern region, risk increased from West to East with proximity of the Dawna Range. Narrow foci of high baseline incidence can be identified for *P. falciparum* in the East, while the risk was diffusely higher for *P. vivax*.

The interaction between the duration of MP activity and the village status was significant when assuming a linear effect of time (p<0.0001 in the final model) or when using spline (p<0.0001 in the final model). As a result, the effect of the duration of MP activity was modelled separately for non-hotspot villages, hotspot villages before MDA, and hotspot villages after MDA.

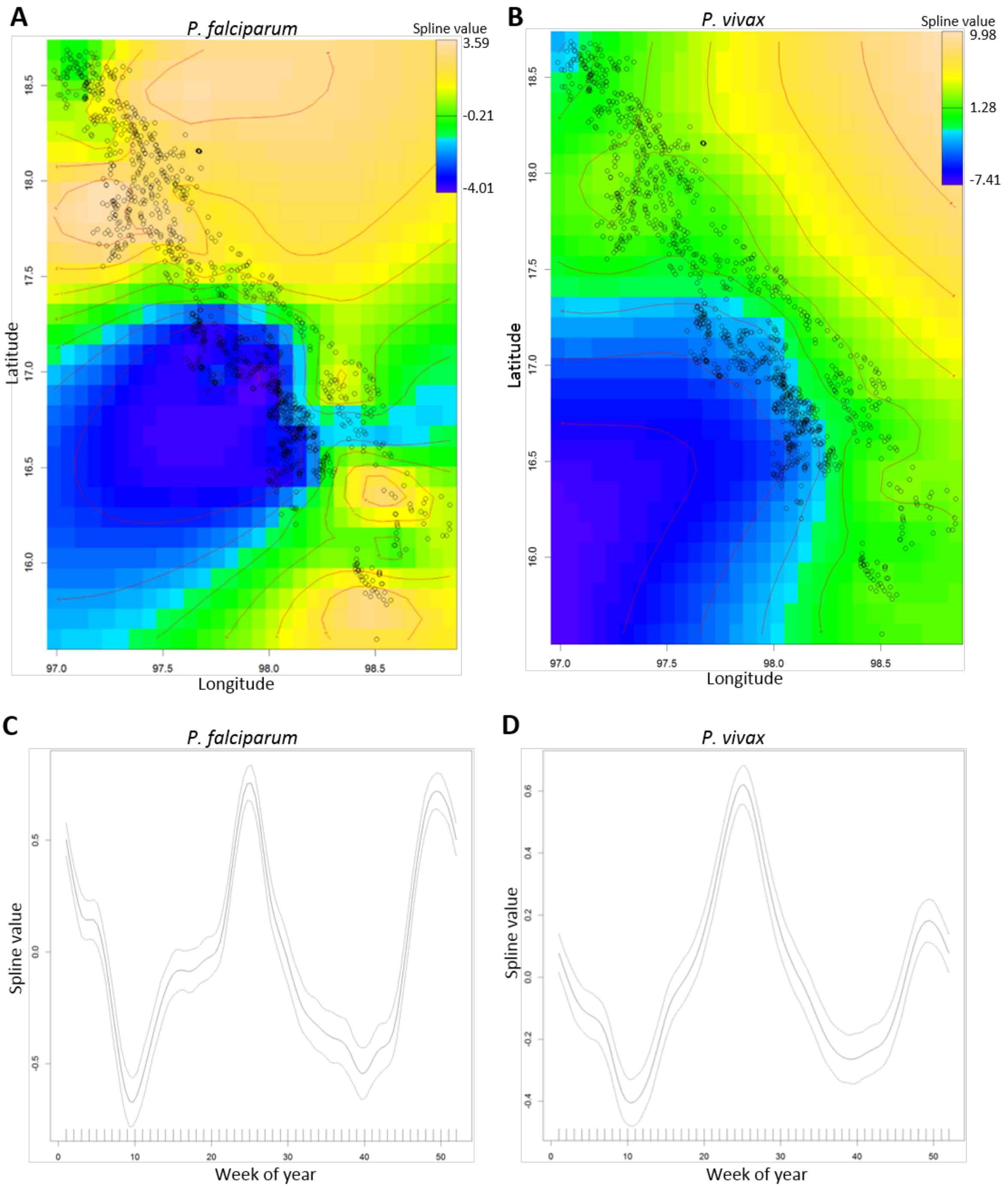

Figure S9: An adjusted heatmap of incidence at MP opening and adjusted seasonal trends for (A+C) *P. falciparum* and (B+D) *P.vivax* incidence. A: Spline values for latitude, longitude providing an adjusted heatmap of *P. falciparum* incidence at MP opening. B: Spline values for latitude, longitude providing an adjusted heatmap of *P. vivax* incidence at MP opening. C: Splines values for the week of year, displaying adjusted seasonality trends for *P. falciparum*. D: Splines values for the week of year, displaying adjusted seasonality trends for *P. vivax*. *Model adjustments include altitude, coverage with MP in the village tract, hotspot and MDA status, MP location in a clinic, plus season (for A and B) and latitude/longitude (for C and D). ~60 villages equipped with MP outside the four targeted townships (in the south and west) are shown to provide more accurate estimates around the limits.*

Supplementary models

The model separating non-surveyed villages from non-hotspots villages confirmed by surveys did not show significant differences with the model presented in Table 2.

The linear assumption for the effect of the duration of MP activity was assessed by comparison with a model where the duration of MP activity was included as a spline function. The linear assumption was acceptable for non-hotspot villages, where a significant decrease was observed, and for hotspots after MDA, where no trend was observed (Figure S10). For hotspots before MDA, the spline showed two main periods: before 6 months and after 6 months of activity. When 2 slopes which were estimated separately in the multivariable model (assuming linear relationship for each period), the IRR obtained were 0.78 (95%CI=0.68-0.89) for the first 6 months and 0.81 (95%CI=0.75-0.87) for the period after 6 months, which did not differ significantly. The small number of MP followed over longer than 18 months after MDA leads to wide confidence intervals around the estimated coefficients.

Table S3: multivariable analysis including MP activity as a spline function of time

Variable		*P. falciparum*			*P. vivax*		
		IRR	95%CI	p-value	IRR	95%CI	
MDA status	No MDA (Non-hotspot)	1	Reference	<0.0001	1	Reference	<0.0001
	Hotspot before MDA	4.16	2.58-6.70		1.84	1.25-2.72	
	Hotspot after MDA	1.69	1.04-2.77		0.72	0.48-1.09	
MP coverage of villages within the village tract (per 10% coverage increment)		0.92	0.89-0.95	<0.0001	0.95	0.92-0.97	<0.0001
MP embedded in a community clinic		1.76	1.16-2.67	0.0079	1.49	1.07-2.7	0.0171
Number of MDA conducted within the village tract excluding the village (per 1 village increment)		0.97	0.93-1.01	0.1571	1.00	0.97-1.04	0.9849
Duration of MP activity in the village		See Figure S10 A-C		<0.0001	See Figure S10 D-F		<0.0001

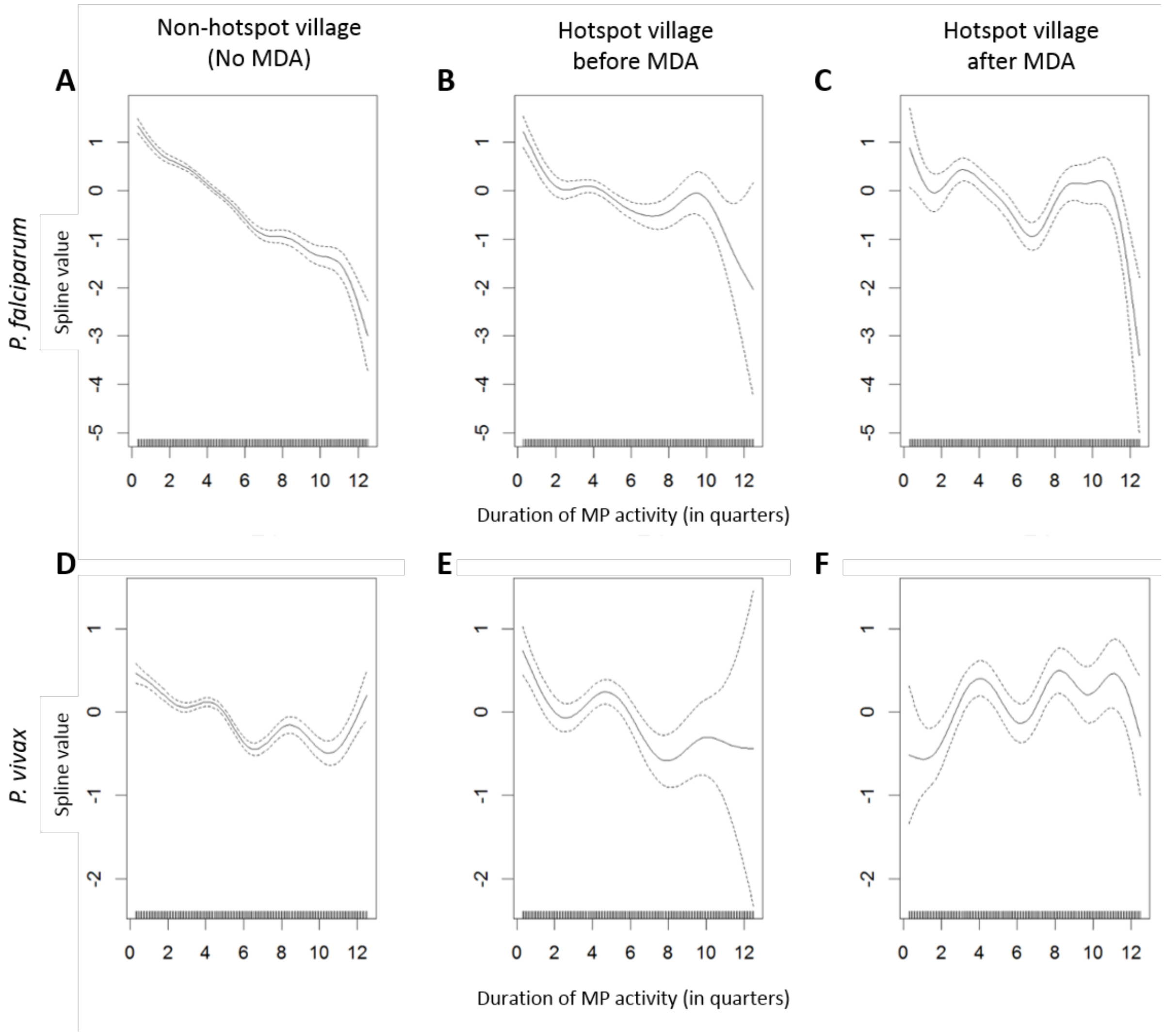

Figure S10: *P. falciparum (A-C)* and *P.vivax (D-F)* incidence over time (in quarters): Spline values (additive coefficient) of multivariable model of incidence relaxing the linear assumption on the impact of the duration of MP opening (Table S3). A: *P. falciparum* incidence in no MDA/Non-hotspot villages. B: *P. falciparum* incidence in hotspot before MDA. C: *P. falciparum* incidence in hotspot after MDA. D: *P. vivax* incidence in no MDA/Non-hotspot village. E: *P. vivax* incidence in hotspot before MDA. F: *P. vivax* incidence in hotspot after MDA.

MALARIA ELIMINATION TASK FORCE GROUP AUTHORS AND AFFILIATIONS

Chiara Andolina[1,2]; Ricardo Aguas[3]; Saw Moe Ang[4]; Ei Phyo Aung[1]; Naw Baw Baw[5]; Saw Aye Be[1]; Saw B'Let[1]; Hay Bluh[1]; Craig A. Bonnington[1]; Victor Chaumeau[1,2,6,7]; Miasa Chirakiratinant[1]; Naw Win Cho Cho[1]; Peter Christensen[1]; Vincent Corbel[6]; Nicholas PJ Day[2,3]; Saw Hsa Dah[1]; #Gilles Delmas[1,2]; Mehul Dhorda[8]; #Arjen M Dondorp[2,3]; Jean Gaudart[16]; Gornpan Gornsawun[1]; Warat Haohankhunnatham[1]; Saw Kyaw Hla[9]; Saw Nay Hsel[1]; Gay Nay Htoo[1]; Saw Nay Htoo[4]; Mallika Imwong[3]; Saw John[10]; Ladda Kajeechiwa[1]; Lily Kereecharoen[1]; Praphan Kittiphanakun[1]; Keerati Kittitawee[1]; Kamonchanok Konghahong[1]; Saw Diamond Khin[10]; Saw Win Kyaw[11]; *#Jordi Landier[1,2,17]; Clare Ling[1,2]; #Khin Maung Lwin[1]; Khine Shwe War Lwin[1]; Naw K' Yin Ma[1]; Alexandra Marie[6]; Cynthia Maung[12]; Ed Marta[10]; Saw Myo Chit Minh[1]; Olivo Miotto[2,3,13]; Paw Khu Moo[1]; Saw Ku Ler Moo[1]; Merry Moo[10]; Naw Na Na[14]; Mar Nay[1]; #François H. Nosten[1,2]; Suphak Nosten[1]; Slight Naw Nyo[1]; Eh Kalu Shwe Oh[10]; Phu Thit Oo[1]; Tun Pyit Oo[1]; #Daniel M. Parker[1, 18]; Naw Eh Shee Paw[1]; Choochai Phumiya[1]; Aung Pyae Phyo[1]; Kasiha Pilaseng[1]; Stéphane Proux[1]; Santisuk Rakthinthong[1]; Wannee Ritwongsakul[1]; Kloloi Salathibuphha[1]; Armon Santirad[1]; Sunisa Sawasdichai[1]; #Lorenz von Seidlein[2,3]; Paw Wah Shee[1]; Naw Paw Bway Shee[1]; Decha Tangseefa[15]; #Aung Myint Thu[1]; May Myo Thwin[1]; Saw Win Tun[1]; Chode Wanachaloemlep[1]; Lisa J White[2,3]; #Nicholas J White[2,3]; Jacher Wiladphaingern[1]; Saw Nyunt Win[10]; Nan Lin Yee[1]; Daraporn Yuwapan[1]
*corresponding author
writing group/coordination team

1.Shoklo Malaria Research Unit, Mahidol-Oxford Tropical Medicine Research Unit, Faculty of Tropical Medicine, Mahidol University, Maesod, Thailand; 2.Centre for Tropical Medicine and Global Health, Nuffield Department of Medicine, University of Oxford, Oxford, UK; 3.Mahidol-Oxford Tropical Medicine Research Unit, Faculty of Tropical Medicine, Mahidol University, Bangkok, Thailand; 4.Burmese Medical Association, Maesod, Tak Province, Thailand; 5.Karen Border Guard Force, Myawaddy Township, Myanmar; 6.Institut de Recherche pour le Développement (IRD), Maladies Infectieuses et Vecteurs, Ecologie, Génétique, Evolution et Contrôle (IRD 224-CNRS 5290 UM1-UM2), Montpellier, France ; 7.Centre Hospitalier Régional Universitaire de Montpellier, Montpellier, France; 8.WorldWide Antimalarial Resistance Network (WWARN); 9.KNU/KNLA Peace Council, Myawaddy Township, Myanmar; 10.Karen Department of Health and Welfare, Maesod, Tak Province, Thailand; 11.Backpack Health Worker Team, Maesod, Tak Province, Thailand; 12.Mae Tao Clinic, Maesod, Tak Province, Thailand; 13.Wellcome Trust Sanger Institute, Hinxton, UK; 14.Klohtoobaw Karen Organization, Kayin State, Myanmar; 15.Faculty of Political Science, Thammasat University, Bangkok, Thailand; 16. Aix-Marseille Univ, APHM, INSERM, IRD, SESSTIM, Hop Timone, Marseille, France; 17. Institut de Recherche pour le Développement (IRD), Aix Marseille Univ, INSERM, SESSTIM, Marseille, France; 18. Department of Population Health and Disease Prevention, University of California, Irvine, U.S.A.
